# Nanocarriers for pancreatic cancer imaging, treatments, and immunotherapies

**DOI:** 10.7150/thno.64805

**Published:** 2022-01-01

**Authors:** Luman Liu, Prakash G. Kshirsagar, Shailendra K. Gautam, Mansi Gulati, Emad I. Wafa, John C. Christiansen, Brianna M. White, Surya K. Mallapragada, Michael J. Wannemuehler, Sushil Kumar, Joyce C. Solheim, Surinder K. Batra, Aliasger K. Salem, Balaji Narasimhan, Maneesh Jain

**Affiliations:** 1 Department of Chemical and Biological Engineering, Iowa State University, Ames, IA.; 2 Department of Biochemistry and Molecular Biology, University of Nebraska Medical Center, Omaha NE.; 3 Department of Pharmaceutical Sciences and Experimental Therapeutics, College of Pharmacy, University of Iowa, Iowa City, IA.; 4 Department of Veterinary Microbiology & Preventive Medicine, Iowa State University, Ames, IA.; 5 Nanovaccine Institute, Iowa State University, Ames, IA.; 6 Eppley Institute, University of Nebraska Medical Center, Omaha, NE.; 7 Fred & Pamela Buffett Cancer Center, University of Nebraska Medical Center, Omaha NE.

**Keywords:** Pancreatic ductal adenocarcinoma, solid tumors, nanoparticles, drug delivery, immunotherapy, tumor microenvironment

## Abstract

Pancreatic tumors are highly desmoplastic and immunosuppressive. Delivery and distribution of drugs within pancreatic tumors are compromised due to intrinsic physical and biochemical stresses that lead to increased interstitial fluid pressure, vascular compression, and hypoxia. Immunotherapy-based approaches, including therapeutic vaccines, immune checkpoint inhibition, CAR-T cell therapy, and adoptive T cell therapies, are challenged by an immunosuppressive tumor microenvironment. Together, extensive fibrosis and immunosuppression present major challenges to developing treatments for pancreatic cancer. In this context, nanoparticles have been extensively studied as delivery platforms and adjuvants for cancer and other disease therapies. Recent advances in nanotechnology have led to the development of multiple nanocarrier-based formulations that not only improve drug delivery but also enhance immunotherapy-based approaches for pancreatic cancer. This review discusses and critically analyzes the novel nanoscale strategies that have been used for drug delivery and immunomodulation to improve treatment efficacy, including newly emerging immunotherapy-based approaches. This review also presents important perspectives on future research directions that will guide the rational design of novel and robust nanoscale platforms to treat pancreatic tumors, particularly with respect to targeted therapies and immunotherapies. These insights will inform the next generation of clinical treatments to help patients manage this debilitating disease and enhance survival rates.

## 1. Introduction

Pancreatic ductal adenocarcinoma (PDAC) is one of the most lethal malignancies of the gastrointestinal tract, with a dismal five-year survival rate of 10%. An estimated 48,220 pancreatic cancer (PC) patients will succumb due to PDAC (8% of all cancer-related deaths), which projects PDAC as the third leading cause of cancer-related deaths in the United States 1. Despite all the efforts, the mortality rate in male PDAC patients has continued to increase by 0.3% annually since 2000, although it has been observed to be stable in female PDAC patients. Current treatment modalities that do not include surgical intervention are largely ineffective and have minimal impact on improving patient survival rates. A majority of PDAC patients are ineligible for surgery due to late diagnosis, early metastasis, and significant local tissue invasion 2, 3. In addition, a lack of biomarkers, high recurrence rate, and chemotherapeutic resistance are other factors that contribute to the high mortality rate of PDAC patients 4-6. To further exacerbate the situation, most pancreatic tumors are poorly responsive to therapeutic approaches due to the highly desmoplastic and immunosuppressive tumor microenvironment (TME) 7-10. The disrupted vascular transport within the pancreatic TME not only influences cellular composition, hypoxia, and tumor metabolic profile but also regulates the response towards systemic therapies 11-13. In particular, pharmacological inhibitors, antibody-based therapeutics, and vaccine-induced immune responses follow a systemic route to reach the TME. Similarly, intrinsic physical and biochemical barriers associated with pancreatic tumors not only affect intratumoral delivery but also compromise the stability and activity of therapeutic agents within the pancreatic TME 12, 14.

Several attempts have been directed towards targeting tumor stroma and vasculature to improve the delivery and efficacy of therapeutic agents towards PDAC 13, 15-18. In this regard, the past two decades have witnessed significant advances in the field of nanotechnology that have introduced not only robust approaches for efficient drug delivery in pancreatic tumors but also provided relevant approaches for the development of vaccine delivery platforms for PDAC 15, 18-21. Considering the challenges associated with the pancreatic TME described above that limit the delivery and efficacy of both chemo- and immunotherapies for pancreatic tumors, advances in nanotechnology-based approaches can play a significant role in overcoming these challenges.

A critical analysis of these advances in nanoscale carrier development, vis-à-vis effective treatments against PDAC, is the main goal of this review. In addition, we also provide an overview of the mechanisms implicated in nanocarrier-based modulation of pancreatic tumor stroma and immune responses directed towards PDAC. Together, the knowledge and insights gained from the analyses herein can set the stage for future developments and next-generation therapies to advance patient health and significantly increase survival rates. The review describes the multiplexed barrier presented by the pancreatic TME to systemic therapies, followed by a summary of various nanoscale delivery vehicles and adjuvants. Next, advances in nanocarrier-mediated delivery of therapeutic payloads for PC are analyzed, and finally, the development of nanocarrier-driven immunomodulatory approaches for PC is discussed.

## 2. Pancreatic tumor microenvironment and therapy resistance

**Figure 1:**
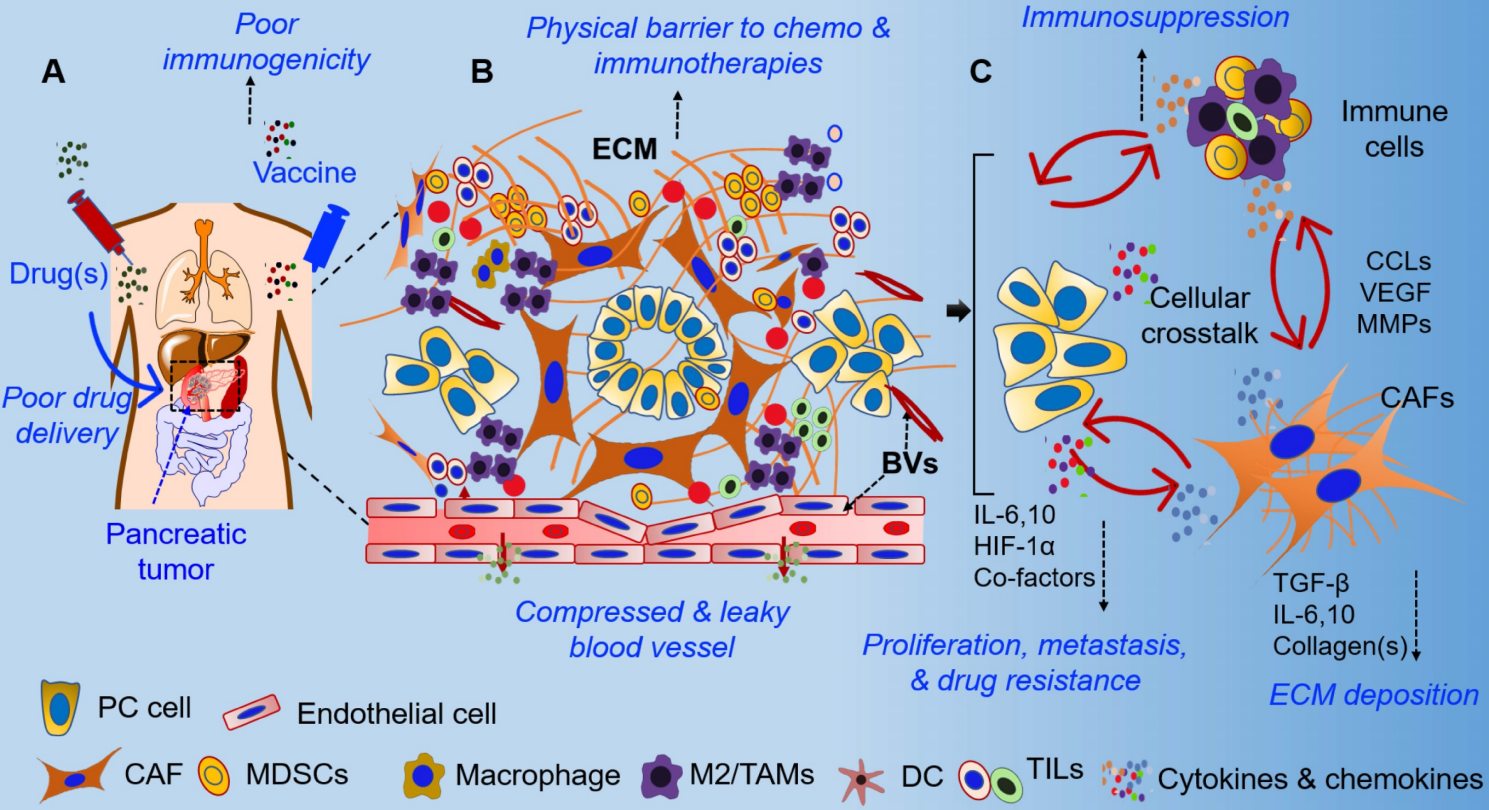
** Therapeutic and immunological challenges in pancreatic cancer. (A)** Therapeutic approaches, including chemotherapies, antibody-based therapeutics, and vaccines, have different challenges related to their delivery and *in vivo* stability. Chemotherapies undergo systemic clearance, metabolize in the liver, and show poor tumor-specific delivery. Similarly, pancreatic cancer is poorly immunogenic, and there is a lack of tumor-specific high-quality antigens to induce a clinically relevant anti-tumor immune response, which causes poor efficacies of vaccine-based immunotherapies in pancreatic cancer. **(B)** Pancreatic tumor microenvironment includes both the physical and biochemical components in the stroma, such as high ECM deposition and disrupted vasculature, which lead to poor drug delivery and interfere in immune infiltration. **(C)** Cellular crosstalk between cancer cells and stromal cell populations leads to various pathological hallmarks of PDAC, including PC progression, metastasis, drug resistance, and immunosuppression. Different cell types of pancreatic tumor microenvironment have been mentioned in the figure (lower panel). Major challenges and hallmarks of PDAC are mentioned in blue color. The colored dots represent various cytokines and chemokines that are present in the pancreatic TME and participate in cellular crosstalk in the local *milieu*. ECM: Extracellular matrix; TAM: Tumor-associated macrophage; CAFs: Cancer-associated fibroblasts; DCs: Dendritic cells.

The extreme resistance of PDAC to chemotherapy, radiation therapy, and immunotherapy is attributed to its complex and obstructive tumor microenvironment. Dense desmoplastic stroma, which is the hallmark of PDAC, is comprised of various cell types, including cancer cells, cancer-associated fibroblasts (CAFs), neurons, tumor endothelial cells, tumor-associated macrophages (TAMs), and other immune cells. These cells are embedded in a collagen-rich extracellular matrix (ECM), which also contains hyaluronic acid, fibronectin, chemokines, cytokines, and extracellular proteases (**Figure 1**) 11, 22. The interaction of tumor cells with various stromal cells along multiple signaling axes directs the evolution of the TME (**Figure 1**). The cellular and acellular components of the pancreatic TME orchestrate biochemical, biophysical, and physiological processes that contribute to therapy resistance. Specifically, the growing tumor cells and excessive collagen induce solid stress and tissue stiffness, leading to the compression of blood vessels and elevated interstitial fluid pressure (IFP). As a result, pancreatic tumors are hypovascular and exhibit decreased perfusion, convection, and diffusion, and, therefore, have impaired delivery of systemic therapies 14, 16. Further, pancreatic tumors are highly heterogeneous in cellularity, stroma composition, and vascularity, and secondary pathophysiological effects such as acidic pH and hypoxia change the tumor metabolic profile and contribute to activation of tumor cell-intrinsic pathways of therapy resistance 11, 12, 23-27. Recent studies have emphasized tumor cellularity as an important determinant of disease progression, epithelial-mesenchymal transition (EMT), metastasis, and therapeutic responses in PDAC patients 28, 29. In particular, high cellularity within the TME of PDAC patients has been reported as a negative prognostic factor. On the other hand, the stromal composition and matrix density in pancreatic TME is a critical determinant of therapeutic response in PDAC 12, 13, 21. Unlike other malignancies, pancreatic tumors are extensively fibrotic (i.e., desmoplastic) and composed of heterogeneous CAFs, which are the major architects of TME in PDAC 30, 31. According to the conventional definition, CAFs have irreversibly activated fibroblast cells that secrete ECM components, including collagen(s), fibronectin, cytokines, and growth factors, and play an important role in tumor progression 32. However, recent studies have further classified CAFs based on the expression of molecular markers, their activation state, and their tumor-promoting or restraining functions 30, 31, 33-35. In terms of ECM deposition, CAFs are the major stromal cell populations that contribute 60-90% of ECM and cause elevated physical stress, a consequence of increased IFP and disrupted vascular function 36-38. Therefore, selective targeting of pro-tumorigenic CAFs might be a potential strategy for normalization of stroma and vasculature in pancreatic tumors and represents an important step towards increasing drug delivery and efficacy in PDAC.

The pancreatic TME is highly immunosuppressive and considered to be unfavorable for immunotherapies in the majority of PDAC patients 7, 8. Recently, next-generation sequencing and next-generation tissue microarray analysis suggested that ~65% of human pancreatic tumors exhibit “immune escape” phenotypes 39. Unsurprisingly, the overall response rate to immunotherapies is poor in PDAC patients, attributed to local tissue stress and vascular disruption in the immunosuppressive TME 8, 40, 41. Besides activated CAFs, various other immune cell populations (including regulatory T and B cells, TAMs, myeloid-derived suppressor cells, and their secreted cytokines) contribute to immunosuppression in pancreatic tumors 42-44. In addition, the TME has been reported to alter the phenotype of infiltrating anti-tumor immune cells to that of “anergic,” “exhausted,” and/or “dysfunctional” phenotypes 45-48. Similarly, myeloid cells, TAMs, and tumor-associated NK cells have been reported to play pro-tumorigenic roles in pancreatic tumors, resulting in poor responses to immunotherapies 49-52. Nevertheless, selective targeting of stromal components, including hyaluronan, collagen(s), CAFs, and stroma-promoting signaling pathways, have been reported to improve the anti-tumor immune response in PDAC 49, 53-56. Recent studies have focused on the use of nanocarriers and targeting for modulation of the stroma to enhance immune infiltration and for the re-activation of immune effector functions in pancreatic tumors 57, 58. For example, targeting hyaluronan synthesis by incorporating an inhibitor in a nanocarrier resulted in ECM remodeling and improved γδ-T cell infiltration 59. Similarly, silencing of retinoic acid-inducible gene 1 (RIG1) by using a selective agonist encapsulated in lipid calcium phosphate (LCP) nanoparticles (NPs) enhanced the anti-tumor effect by silencing BCL2, which enhanced apoptosis 60. This was positively correlated with increased Th1 proinflammatory cytokine levels, infiltration of more CD8^+^ T cells compared to regulatory T (Treg) cells, and the presence of more M1 over M2 macrophages. Correspondingly, a decrease in regulatory B cells in the NP-RIG1-agonist treatment group also indicated the immunomodulatory effects of the nanoformulation 60. Further, gene delivery using the same LCP nanoplatform showed selective delivery of a plasmid encoding relaxin into metastatic liver tissues. Interestingly, forced expression of relaxin not only reduced the metastatic burden but also altered stroma and immune milieu in a liver metastasis model of PDAC 61. Several other nanocarrier platforms have been demonstrated to effectively target pancreatic tumors and deliver immunomodulatory agents. These include trapping of IL10 and CXCL12 by using lipid protamine DNA NPs loaded with the trapped gene 62, use of oxaliplatin (OX) with encapsulated siIDO-1 (indoleamine 2,3, dioxygenase-1) 63, mesoporous silica NPs (MSNs) loaded with glucose oxidase, cancer cell surface as camouflage with anti-PD1 therapy 64, and NPs loaded with standard chemotherapies 65-67. These nanocarriers were demonstrated in various PDAC models to modulate stroma, increase the presence of effector immune cells, and decrease the immunosuppressive cytokine milieu. There is ample evidence to show that nano-driven strategies are suitable for drug delivery and effective in stromal modulation and in potentiating immunotherapy-based approaches in PDAC. The various physical, biochemical, and immunological changes in the pancreatic TME due to treatment with nano-based formulations are summarized in **Figure 1**.

In contrast, pharmacological inhibitors and antibody-based therapeutics differ in structure, function, and physiological stability and, therefore, need various approaches to improve their pharmacokinetics and pharmacodynamics. For example, vaccine formulations need sustained antigen release for durable immune responses, whereas chemotherapies need increased tumor availability and slower clearance. On the other hand, antibody-based therapeutics require improved stability *in vivo* to evoke effective responses. Nano-driven strategies could be used to enhance stability under physiological conditions and sustain the bioavailability of therapeutic agents, as detailed in Section 4. Thus, nanotechnology provides clinically relevant platforms that reduce stromal hindrance, enhance drug delivery and stability, and improve immune cell infiltration, as well as improve the efficacy of immunotherapy-based approaches by their immunomodulatory functions in PDAC (as described in **Section 4**).

## 3. Nanocarrier-based delivery of therapeutic, imaging, and theranostic payloads for PDAC

### 3.1. Nanoscale drug delivery vehicles and adjuvants

Current therapeutic modalities for cancer treatments are comprised of surgery, chemotherapy, radiotherapy, immunotherapy, or rational combinations. Thereof chemotherapy is the standard-of-care treatment and is the longest-serving modality for treating various cancers, including PDAC. However, direct administration of drug payloads often causes compromised delivery, systemic toxicity, and severe side effects. In addition, poor drug pharmacokinetics (i.e., solubility, stability, and metabolism) result in limited biodistribution, low therapeutic efficacy, and inadequate responses. Alternatively, immunotherapy is emerging as a promising therapeutic option for cancers with improved responses against primary and metastatic tumors 68. Despite these advances, direct delivery of immunotherapeutic agents (e.g., cytokines, checkpoint inhibitors, etc.) suffers from suboptimal pharmacokinetics and susceptibility to degradation, resulting in adverse effects 69, 70.

**Figure 2:**
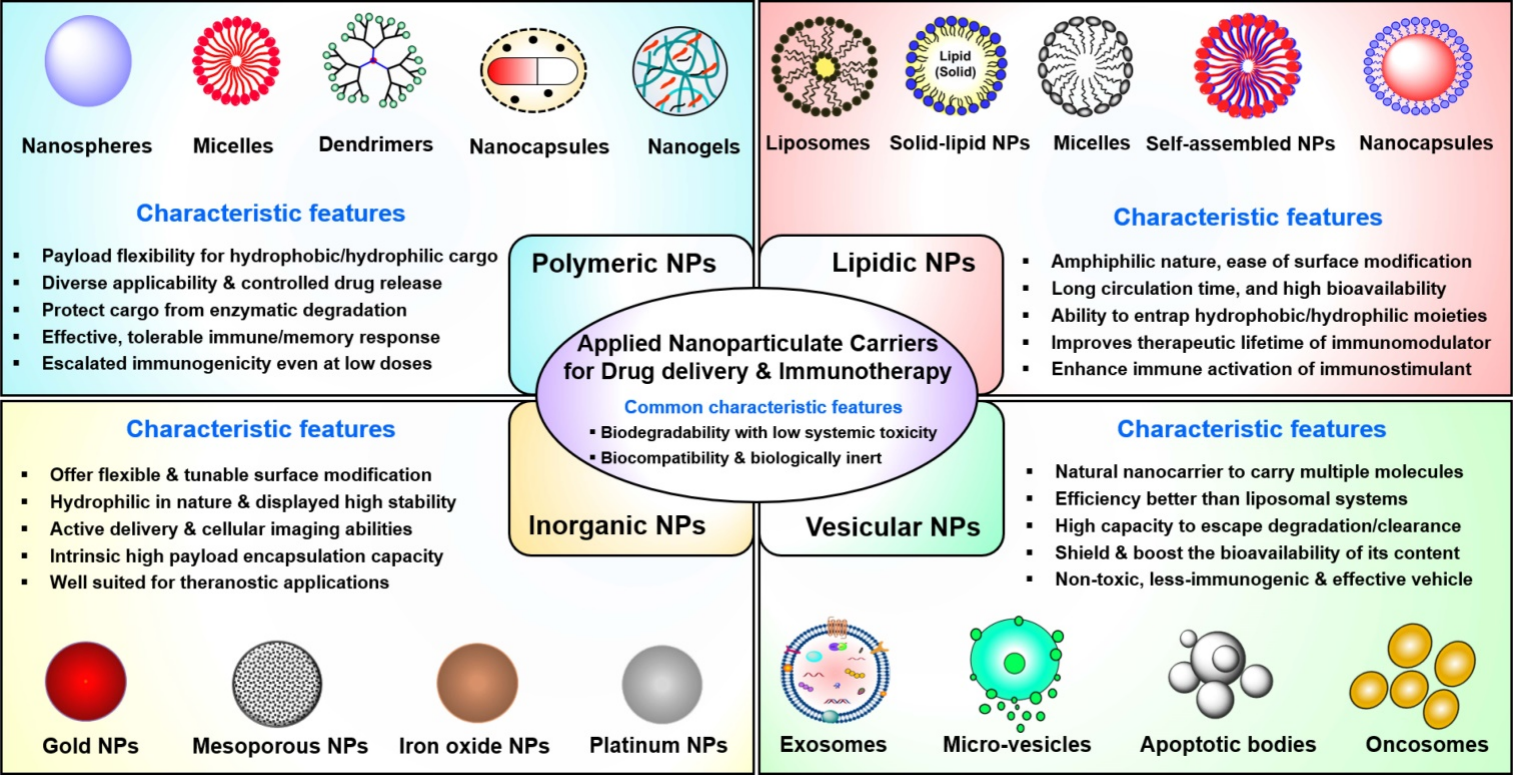
** Engineered nanocarriers for PDAC drug delivery and immunotherapy.** This figure provides schematic illustrations of major types and multiple subtypes of nanocarriers and their characteristic features that have been employed for drug/theranostic payload delivery and immunotherapy against PDAC. Clockwise from left: Polymeric, lipidic, nano/micro vesicle-based, and inorganic materials-based nanocarriers. The schematic structure of each nanocarrier subtype is depicted in the top and bottom rows. The most commonly observed features of each nanocarrier class are mentioned in the middle. NPs: Nanoparticles.

Furthermore, non-specific interactions of soluble immunotherapeutic payloads with immune cells, nucleases, and proteases not only reduce immuno-stimulatory responses but also contribute to immune-related adverse effects. Thus, there is an urgent need to develop effective delivery platforms to transport therapeutic/immunological payloads to their target cells and/or tissues, along with minimal exposure to their biological environment and reduced side effects. Previously, various nanomaterial-based carriers (i.e., nanocarriers) have been designed to overcome the issues outlined above, whereby therapeutic payloads are conjugated to or entrapped within biocompatible nanomaterials to enhance their ability to overcome sequential biological barriers associated with a TME 71-74. The benefits of this approach include protection of payloads from degradative agents, minimization of non-specific interactions, enhanced biological stability (i.e., prolonged circulatory half-life), increased bioavailability of payloads, dose sparing, and enhancement of specific tissue targeting 75, 76. The following sections are focused on the chemistries, characteristic features, advances, and clinical applications of nanocarriers (**Figure 2**) in cancer therapeutics, including PDAC. This section also discusses how different nanocarriers have been used to deliver therapeutic payloads for PDAC treatment, including chemotherapeutic and nucleic acid drugs and imaging agents. Various types of FDA-approved or clinical-stage nanomedicines used for small molecule drug delivery to PDAC are shown in **Table 1**.

#### 3.1.1. Polymeric NPs

Polymeric NPs are well-studied as nanocarriers for drug delivery and immunotherapy 77. Polymeric NPs allows for a wide range of conjugation and encapsulation options accompanied by excellent biocompatibility profiles and effective delivery at the desired site(s) of action 71. Polymeric NPs can protect encapsulated payloads from degradation and enhance their bioavailability to tumors and other tissues by delivering maximum dose via the enhanced permeability and retention (EPR) effect 78. Compared to liposomes, polymeric NPs show enhanced stability and resistance to drug leakage, while smaller-sized NPs have been repNanoparticle-based delivery for PCorted to lengthen the half-life of therapeutic cargos in circulation, reduce their degradation, and provide sustained release, which would enhance the accumulation of the cargo in the target tissue 79. Additionally, the ability of polymeric NPs to adsorb or be coated with targeting ligands, combined with their inherent adjuvant properties, make them attractive candidates for induction of tumor-specific immunity (Section 4).

Biodegradable polymers (both natural and synthetic) have been widely used to synthesize NPs 80. Among synthetic polymers, multiple types of commercial biodegradable polymers, including polyethylene glycol (PEG), polyesters [such as poly(lactic-co-glycolic) acid (PLGA)], and polyanhydrides [(based on monomers such as sebacic anhydride (SA), 1,3-bis(*p*-carboxyphenoxy propane) (CPP), 1,6-bis(*p*-carboxyphenoxy hexane) (CPH), and 1,8-bis(*p*-carboxyphenoxy hexane)-3,6-dioxaoctane (CPTEG)] have been investigated as nanocarrier platforms 81-84. PEG has been widely used in drug delivery 85. PEG can be used to deliver hydrophobic small molecule drugs by improving solubility compared to the drug alone. PEG is also used as a coating on other types of NPs. The process of attaching PEG to another drug or molecule is referred to as PEGylation. PEGylation reduces unwanted immune recognition, resulting in longer circulatory half-lives of small molecule drugs, which is beneficial when delivering chemotherapeutics. For example, PEGylation contributed to the success of both Doxil^®^ and Genexol^®^ 71. Various types of NPs, including gold 86, 87, polymeric, and lipid NPs carrying small molecule drugs (e.g., doxorubicin) for PDAC, have been PEGylated to improve their pharmacodynamic characteristics 88, 89. PLGA has been widely used as a nanocarrier for drug delivery because of its adaptability, suitability, and ease of manipulation with respect to its chemical and physical properties such as hydrophobicity/hydrophilicity, molecular mass, and crystallinity, which can be modified by changing the monomer ratio, terminal group chemistry, size and net surface charge 90. The chemical properties of PLGA allow hydrolytic degradation by de-esterification. For example, polylactide and polyglycolide are composed of monomeric components that are easily metabolized by the body, and their rates of degradation as well as physicomechanical properties are tunable over a wide range by using polymers of varied molecular weights and molar ratios 91.

Biodegradable polyanhydride-based NPs have also been widely studied as drug delivery vehicles 92-101. Copolymers based on SA, CPP, CPH, and CPTEG display tunable surface erosion kinetics (controlled by the hydrophobicity, which in turn depends upon the copolymer composition), leading to highly controlled and sustainable drug release 102. These materials are easy to functionalize because of their carboxylic acid end groups, which has led to targeted delivery approaches that help navigate tough-to-penetrate biological barriers such as tumors, bacterial membranes, and the blood-brain barrier 103-107. Polyester NP-encapsulated chemotherapeutic drugs have been broadly investigated 108, 109. Among them, poly(L-glutamic acid) (PGA), PTX (Xyotax) 110, and PGA-camptothecin (CT-2106) 108 are FDA approved or are in clinical trials as anti-cancer nanomedicines. PLGA NPs have also been used in a targeted approach to deliver Taxol (PTX) to PC cells in both *in vitro* and *in vivo* settings 111. In these studies, PEG blocks were used to increase the “stealth” of the NPs. Release studies showed that over 90% of Taxol was released within one week. This targeted delivery approach showed decreased tumor volume compared to controls *in vivo*. Multi-functional gene therapy platforms based on poly[oligo(ethylene glycol) methyl ether methacrylate] NPs combine shielding (provided by the short PEG block) and RNA binding capability (provided by the cationic PDEAEM moiety) with enhanced retention, high RNA loading, and increased cellular uptake, all of which translated to NP accumulation at the tumor site and growth inhibition 112. The design of siRNA-adjuvanted GEM-based PC treatment involved the use of a cationic ε-polylysine copolymer NP core, enabling efficient loading of HIF1α siRNA and GEM. The NPs were further coated with a PEGylated lipid bilayer to prevent rapid degradation of the payload and avoid particle aggregation. The synergistic antitumor effect was demonstrated in both a subcutaneous xenograft tumor model and an intravenously administered orthotopic tumor metastasis model 89. The same group also designed RRM2 siRNA-adsorbed 1,2-dioleoyl-3-trimethylammonium-propane (DOTAP) cationic liposomes loaded with GEM, which were shown to significantly sensitize cancer cells to GEM treatment in a subcutaneous PANC-1 murine model 113.

#### 3.1.2. Micelles, dendrimers, and nanogels

Polymeric micelles, which are self-assembled amphiphilic core-shell particles, are efficient in delivering highly hydrophobic drugs. Bioconjugation or physical entrapping of the hydrophobic drug into micelles can provide minimal drug leakage, maximizing drug solubility and half-life in blood circulation and improving delivery 114, 115. Block copolymers are most often used to produce micelles because of their amphiphilic properties, which allow the formation of a hydrophobic core and hydrophilic outer portion. Micelles work well to deliver hydrophobic drugs because the drugs are trapped in the hydrophobic core. Hydrophilic drugs can also be delivered using micelles when they are associated with the outer portion of the micelle.

Kumar *et al.* developed a block copolymer micelle based on PEG block-poly(2-methyl-2-carboxyl-propylene carbonate-graft-dodecanol-graft-tetraethylenepentamine) to deliver Vismodegib (small molecule hedgehog pathway inhibitor) and microRNA (miRNA) to treat PDAC in an orthotopic murine model 109. The elimination half-life of the drug and biodistribution of Vismodegib was improved using these micelles. Micelles assembled from cationic polymers can be complexed with nucleic acids and be readily internalized by target cells. By bioconjugation or insertion of functional moieties into the multiblock polymer, micelles can improve targeting and delivery of multiple payloads simultaneously to pancreatic tumors. For example, Pittella *et al.* designed PEGylated calcium phosphate (CaP) hybrid micelles that could deliver siRNA to PC 116. In the micelle design, a PEG layer shield, a CaP nanocore for polymer binding, and a pH-sensitive cis-aconitic amide incorporated endosome-disrupting copolymer were integrated to enable pancreatic tumor targeting and pH-responsive endosomal escape of siRNA. The micelles were tested in a transgenic murine model and shown to improve siRNA accumulation at the tumor site (demonstrated by luciferase gene silencing). In another study, micelles were prepared for the co-delivery of DTX and Atg7 siRNA to inhibit PC cell autophagy and sensitize cancer cells to chemotherapy. Micelles synthesized from a Pluronic® P123 backbone and integrin-binding iRGD were shown to target nude mouse PANC-1 xenograft tumors and released the trapped DTX and siRNA. Increasing micelle stability in blood circulation is another strategy to improve anti-cancer efficacy 117. Uchida *et al.* prepared micelles attached to a cholesterol moiety to increase blood circulation stability by hydrophobic interaction. The micelles loaded with mRNA encoding anti-angiogenic protein sFlt-1 were shown to be therapeutically beneficial in a BxPC-3 pancreatic tumor model that shares histopathological features with human PC 118. Chemoresistance and invasion of pancreatic cancer stem cells (CSCs) mediated by miRNA has been proposed as a mechanism for PC drug resistance and frequent recurrence. Micelles conjugated with GEM and a miRNA-205 mimic were tested against CSCs. Co-delivery was demonstrated to be synergistic in reducing cancer growth in both GEM-resistant CSCs and xenograft pancreatic tumors 119.

Polymeric nanogels are three-dimensional (physically or chemically) crosslinked networks with high water content. As potential carriers, polymeric nanogels can improve stability and provide longer retention and greater loading capacity of the therapeutic payload 120. In recent work by members of our team, temperature- and pH-responsive pentablock copolymers, consisting of a temperature-responsive Pluronic^®^ F127 middle block and pH-responsive poly(diethylaminoethylmethacrylate) (PDEAEM) end blocks were developed for dual delivery of miR-345 and GEM 121. Recent reports have also discussed the use of nanogels as targeted nanomedicines to increase treatment effectiveness and improve outcomes of PC therapy 122, 123.

Dendritic polymers have also been used as delivery systems owing to their multivalent characteristics, defined molecular weight, monodisperse size, and water solubility. Further, the globular structure of dendrimers with an available internal cavity (central core) and modifiable surface functionality makes them attractive vehicles for payload delivery 124. Both *in vitro* and* in vivo* studies testing therapeutic efficacy of doxorubicin (DOX)-loaded dendrimeric polymer compared to i.v. delivered DOX revealed a 10-fold improvement in cellular uptake and a 9-fold reduction in cellular toxicity 125.

#### 3.1.3 Lipidic NPs

Lipidic NPs are well-established and easy-to-produce nanocarrier delivery systems. Liposomes, the first NP platform to be applied in medicine, are composed of nanosized synthetic vesicles consisting of one or multiple spherical shell bilayers, encompassing an aqueous core 126. Compared to some polymeric NPs, lipidic NPs are less toxic and exhibit higher biocompatibility because their structural components display similarities with plasma membrane lipids and human cholesterol. Lipidic NPs are classified as liposomes, solid-lipid nanoparticles (SLN), phospholipid micelles, or nanocapsules 127. Liposomes possess the unique characteristic of loading hydrophobic drug moieties within the shell layer while entrapping hydrophilic payloads within the aqueous core to protect them from degradation and metabolism. Compared to free drugs, liposomes can help in modifying pharmacokinetics and biodistribution of encapsulated drugs by augmenting drug circulation time, tumor exposure, and retention, thereby boosting the overall therapeutic effect on cancer cells 128, 129. Several stimuli-responsive liposomes have been developed to achieve target-selective delivery of the entrapped drug. A change in temperature/pH (e.g., endosome) can trigger the intracellular release of drugs and improve the therapeutic efficacy of lipidic nanomedicines 130-132. Similarly, SLNs also show attractive physicochemical properties, high biocompatibility, and the capability to deliver hydrophobic compounds 133.

With multiple continuing efforts for developing potential cancer nanotherapeutics, several liposomal drug products are available in the market, including Doxil^®^, DaunoXome^®^, Depocyt^®^, Myocet^®,^ and others. Resembling liposomal characteristics, SLNs have shown attractive physicochemical properties, high biocompatibility, and the capability to deliver hydrophobic compounds. SLNs offer the precise release of the immune reagents, mitigate off-target CTL response, and effectively harness immune responses by activating either a humoral or cellular immune response against cancer cells 134. Stimuvax, Tecemotide, and sHER2+AS15 are notable examples of liposome-based cancer nanovaccines that have progressed through phase-II/III clinical trials to treat PDAC, along with other carcinomas 130, 132.

#### 3.1.4. Extracellular vesicle-based NPs

Extracellular vesicles (EVs) are cell-derived, nanosized membrane vesicles. Based on their size and biogenesis processes, EVs are subdivided into four subtypes: exosomes (30-100 nm), microvesicles (50 nm-1 μm), apoptotic bodies (20 nm-5 μm), and large oncosomes (1-5 μm) 135. These subtypes differ in their origin, composition, and biochemical properties. As natural transporters, EVs have gained considerable scientific interest in cancer therapeutics because of their ability to shuttle biomolecular cargoes between cells 136, 137. Exosomes have been demonstrated to establish a pre-metastatic niche in PDAC and dictate metastatic organotropism 138, 139. Due to their natural origin (via biogenesis) and ability to target specific organs, EVs have multiple advantages over conventional drug delivery systems, including high biocompatibility, prolonged stability, ability to pass through natural barriers, intrinsic cell targeting, reduced toxicity, and low immunogenicity. To date, EVs have been shown to deliver proteins, nucleic acids, small molecules, drugs, and CRISPR/Cas9 systems 140. Among these membrane-derived vesicles, exosomes are the most applied EVs in cancer theranostics due to their high versatility 141. Exosomes from diverse cellular origins, including tumor cells, fibroblasts, macrophages, and mesenchymal stem cells (MSCs), have been loaded with therapeutic cargoes, including chemotherapeutic drugs and siRNA, for delivery to PC cells. Compared to liposomes, exosomes contain cell-of-origin-derived transmembrane-anchored proteins, which can regulate their clearance from phagocytosis. A recent study demonstrated that exosomes could be engineered to prevent their clearance via phagocytosis and inhibit KRAS by selectively delivering short interfering (siRNA) or short hairpin RNA (sh RNA) to PC cells 142. It was observed that CD47 on the exosome regulates their clearance by circulating monocytes. Exosomes isolated from CD47-knockout mouse fibroblasts and loaded with siRNAs or shRNA targeting mutant Kras^G12D^ efficiently delivered cargo to orthotopically implanted and autochthonous pancreatic tumors and resulted in decreased tumor growth and metastasis, resulting in improved survival 142. Paclitaxel-treated immortalized MSCs were found to incorporate, package, and release the active drug in the exosomes. The drug-loaded exosomes were demonstrated to be taken up by PC cells *in vitro* and inhibit their growth 143. Similarly, exosomes isolated from bone marrow MSCs were loaded with gemcitabine monophosphate by reversible electroporation and paclitaxel by sonication. The dual drug-loaded exosomes exhibited superior penetration, anti-tumor, and anti-stromal effects on orthotopic pancreatic tumors as compared to the clinically approved Gem+Nab-paclitaxel (Abraxane) or GEM-alone loaded exosomes 144. Macrophage-derived exosomes have also been examined for packaging and delivering chemotherapeutic agents to PC cells. Exosomes isolated from a human macrophage cell line THP-1 and loaded with GEM and Deferasirox, an oral iron chelator, effectively inhibited the proliferation of GEM-resistance PC cells in 2D and 3D cultures *in vitro* 145. Recently, exosomes isolated from Panc-1 PC cells were loaded with GEM either by direct incubation with the drug or sonication 146. These GEM-loaded autologous exosomes resulted in a significant decrease in tumor volume and prolonged survival of mice with no evidence of non-target tissue toxicity as compared to the free drug 146. The utility of exosomes as vectors for delivering therapeutic agents for PC has been described in detail in a recent review article by Oliveria *et al.* 147.

#### 3.1.5. Inorganic NPs

Inorganic NPs have been widely applied to the treatment and diagnosis of cancer. Compared to polymeric NPs, inorganic NPs can be manufactured with more defined morphology, size, and surface chemistry. Based on the electrochemical and magnetic properties of the materials, techniques such as magnetic resonance imaging, surface plasmon resonance spectroscopy, and surface-enhanced Raman scattering spectroscopy provide characterization with high resolution and low tissue background 148. A variety of inorganic NPs has been employed in nanotherapeutic applications 149. Among them, gold nanoparticles (AuNPs), MSNs, and iron oxide NPs have emerged as leading candidates because they are biologically inert and flexible to surface modification. Additionally, their hydrophilic nature, resistance to microbial growth, high stability, and low toxicity provide added advantages. AuNPs have emerged as a potential tool for anticancer therapy due to their characteristic visibility and ease of functionalization. MSNs are also promising payload carriers with good biocompatibility and distinct porous architecture, which enables high cargo loading efficiency 150. Magnetic NPs, based on superparamagnetic iron oxide (SPION), possess high magnetization and moderate biocompatibility, and have shown great promise in cancer therapeutics 151. SPIONs allow the transport of therapeutic cargos and other payload moieties, i.e., imaging probe and radiotherapy payloads 152.

Various AuNP-based conjugates are being evaluated *in vitro* and in preclinical animal model studies to deliver routinely used chemotherapeutic drugs, such as docetaxel (DTX) and 5-fluorouracil 153. Two AuNP-drug nanoconjugates, namely, AuraLase and NU0129, are in clinical trials for lung cancer and glioblastoma therapy, respectively 154. Moustaoui *et al.* used PEGylated Au(III) NPs to deliver DOX to PDAC cells *in vitro* and demonstrated that DOX release was pH-dependent 86. Studies with drug-loaded SPION showed enhanced cellular permeability and augmented tumor-targeting abilities via surface peptide interactions, supporting their utility for cancer treatment. The FDA has already approved magnetic SPION-based formulations (e.g., Feraheme^®^, Feridex I.V.^®^, and Gastromark^®^) as magnetic resonance imaging (MRI) contrast agents. However, investigations concerning theranostic applications of SPIONs are still at the preclinical stage because key issues related to magnetic NPs are yet to be addressed 155. Lee *et al.* developed pH- and lysozyme-dependent iron oxide NPs for the release of GEM, using orthotopic tumor models as well as MiaPaCa-2 cells 156 The NPs showed a statistically significant reduction in tumor growth in the mouse models and provided superior imaging capabilities in MRI.

A nanocarrier for the dual delivery of siRNA and drug was prepared from graphene quantum dots (GQDs) by Yang and co-workers 157. The nanocarrier was functionalized with biodegradable charged polyester vectors to encapsulate siRNA targeting KRAS mRNA. The resulting GQDs integrated photothermal therapy, siRNA release, and enhanced DOX efficacy against a MiaPaCa-2 PC cell line. AuNPs have also been used as nanocarriers for siRNA targeting nerve growth factors in PC. For example, novel fluorescent gold nanoclusters were characterized for size, siRNA release, and gene silencing performance and shown to significantly inhibit tumor growth and decrease neurite density 158. Another study demonstrated the dual delivery of GEM and miRNA-21 inhibitor (miR-21i) using dendrimer-entrapped AuNPs 159. The internal cavities and terminal amine groups of the dendrimer provided the capacity for GEM loading and miR-21i electrostatic compression. The co-delivery of miR-21i and GEM aided by ultrasound-targeted microbubble destruction was tested in a xenograft PC model. Most inorganic nanomaterials offer reasonable biocompatibility, moderate stability, and unique diagnostic and therapeutic opportunities that organic or traditionally used NPs cannot offer. Despite these advantages, inorganic NPs have limited success in entering clinical trials due to their low solubility and concerns related to their toxicity, biodistribution, and subsequent clearance. Recent examples showed that combining the potential of inorganic NPs with organic materials by functionalizing/coating biocompatible materials to the surface of inorganic NPs can provide avenues for the use of inorganic NPs scaffolds in the clinic 160-162.

#### 3.1.6. Natural NPs

Natural polymers such as albumin, chitosan, heparin, and others have been formulated as NPs to deliver therapeutic drugs, proteins, and oligonucleotides. These natural polymers are particularly attractive for drug delivery owing to their non-toxic, non-immunogenic, and biodegradable properties 80. For example, albumin-based NPs provide multiple benefits, including high binding capacities for both hydrophobic and hydrophilic drugs, relatively facile preparation, and their ability to be specifically modified to facilitate targeted delivery 163, 164.

Thiolated type B gelatin NPs were used to deliver GEM to PC *in vitro* and *in vivo* 165. The IC_50_ value in PANC-1 cultures decreased when gelatin NPs were used. Tumor growth reduction was also observed during *in vivo* studies. Human serum albumin NPs loaded with PTX (i.e., Nab-PTX), combined with GEM, are an FDA-approved treatment for PDAC 166. This Nab-PTX-GEM was the first combination therapeutic to include GEM that increased patient survival time 163. In addition, the hydrophobicity of Nab-PTX was decreased compared to PTX, which led to better solubility in the bloodstream and improved pharmacokinetics 167. Nab-PTX-GEM combination therapy has also shown therapeutic efficacy as a first-line treatment for metastatic PDAC by improving overall response rate and survival compared to GEM alone 163, 168. Additionally, numerous phase I, II, and III clinical trials are ongoing for Nab-PTX-GEM treatments combined with radiotherapy and other drugs 163. Nano-liposomal irinotecan (Onivyde) is being used in the treatment of PDAC patients. The liposomal formulation of irinotecan led to increased cellular uptake compared to free irinotecan 169. In addition, lipid NPs encapsulating GEM were used in an *in vitro* study on BxPC-3 spheroid cultures 170. The NPs were responsive to the hypoxic tumor microenvironment by reducing the lipid, which then released GEM. Other liposomal-drug products that are commercially available in the market include Doxil^®^, DaunoXome^®^, Depocyt^®^, and Myocet^®^.

#### 3.1.7. Hybrid NPs

Built upon the advantages of distinct nanoparticular platforms, hybridization is another strategy to incorporate two or more nanomaterials to overcome multifaceted challenges 171. Gao *et al.* produced hollow, biodegradable mesoporous organosilica NPs, which are pH-sensitive to the more acidic microenvironment of pancreatic tumors, and the NPs effectively released the drug within the tumor 172. This nano-system showed controlled delivery of both GEM and pirfenidone in both *in vitro* and *in vivo* studies. In addition, ultrasound-triggered microbubble destruction was used to increase penetration into the tumor tissue. Li *et al.* produced lipid-polymer hybrid NPs to deliver FOLFIRINOX to pancreatic tumors 173 using a layer-by-layer approach with a polymer core and a PEGylated lipid shell. This NP formulation showed good stability in serum and decreased side effects in *in vivo* studies compared to free FOLFIRINOX. AuraLase, a silica-gold nanocomposite, is currently in clinical trials for thermal ablation therapy for solid/metastatic lung tumors 154.

### 3.2. Nanoparticle-based molecular imaging and theranostic probes for PDAC

Imaging is an integral component of the diagnosis and management of PDAC patients. Among various imaging modalities employed, multidetector computed tomography (CT) angiography is highly sensitive and the most preferred method for initial diagnosis, staging, and resectability assessment 174 due to its widespread availability and low cost. Magnetic resonance imaging (MRI) has comparable sensitivity in staging PDAC, and magnetic resonance cholangiopancreatography (MRCP) enables detailed evaluation of the biliary and pancreatic ductal system 175. While MRI is not as widely used as CT for initial diagnosis, it is more efficient in detecting small tumors, metastatic lesions in liver peritoneum and lymph nodes (LN), and identifying malignant cystic lesions of the pancreas 175, 176. Endoscopic ultrasound (EUS) is highly sensitive in detecting small tumors that are often missed by other imaging modalities, and it also provides an opportunity to collect samples (fine needle aspirates) for cytological or biomarker analysis to facilitate the most conclusive diagnosis 177. Metabolic PET imaging, which relies on the differential uptake of ^18^F-labeled fluoro-deoxy glucose (FDG) by rapidly growing tumor cells, enables whole-body imaging to detect both primary tumors and metastasis and is used alone or in combination with CT and MRI for evaluating the response to therapy in PDAC patients 178, 179. The principles, utility, and current status of various imaging modalities are elegantly reviewed in several recent articles 178, 180-182. Imaging modalities like abdominal ultrasound utilize microbubbles as contrast agents, which have been functionalized by targeting molecules to facilitate molecular imaging. Jugniot *et al.* 183 have comprehensively reviewed the current clinical and preclinical status of targeted microbubbles for PC. A detailed discussion on the subject is beyond the scope of the current review.

Recently, nanoparticles have been engineered to deliver imaging agents alone or in combination with chemotherapeutic drugs and used for imaging or theranostic applications, respectively **(Table 2)**. Several multi-functionalized NPs have been demonstrated to be capable of delivering multiple imaging probes to counter the limitations of single molecule-based imaging modalities to augment image resolution, enhance temporal resolution, and improve tissue penetration and probe sensitivity 184.

Various NPs-imaging probes based on iron oxide, carbon oxide, inorganic metal NPs, and liposomes have been evaluated to deliver imaging agents for diverse imaging modalities, including MRI, CT, PET, and SPECT for PDAC 185, 186. These have been elegantly reviewed in several recent articles 185, 187**.** Zhao *et al*. developed a multimodal (MRI, CT, and PAI) contrast probe using gold nanorod-silica core-shell NPs layered with gadolinium oxide (AuGR-SiO2-Gd). *In vitro*, AuGR-SiO2-Gd NPs exhibited significantly more enhancement in MRI contrast than Gadvovist, a commercial MRI agent, and higher X-ray attenuation, compared to the commonly used contrast agent Visipaque (Iodixanol) on agarose gel phantoms. *In vivo*, AuGR-SiO2-Gd NPs revealed a positive contrast in MRI and a negative contrast within the tumor area in genetically engineered mice in CT and photoacoustic imaging (PAI) 188. The utility of conjugating radiolabeled anti-TAA with AuNPs for PET imaging of pancreatic tumors has recently been demonstrated 189. Fully humanized, anti-CA 19.9 mAb conjugated to p-isothiocyanatobenzyl-desferrioxamine (p-SCN-DFO) to chelate a PET-emitting radionuclide (^89^Zr) was subsequently attached to activated Au-NPs. Radiolabeled mAb-AuNPs allowed for efficient detection of orthotopic pancreatic tumors and established the utility of depleting the mononuclear phagocyte system for reducing the non-specific hepatic uptake of nanoparticles. NP-based nanoprobes have also been developed to differentiate tumors from uninvolved healthy tissue for surgical navigation. Qi *et al*. synthesized hyaluronic acid (HA) NPs encapsulating near-infrared (NIR) dye-indocyanine (ICG), which allowed improved discrimination of primary orthotopic tumors from the healthy pancreas and better detection of splenic metastasis as compared to free ICG 190. Other nano-imaging agents based on various imaging agents and NP compositions that have been evaluated in pancreatic cancer are summarized in **Table 2**.

Theranostic NPs have also been evaluated in several studies for targeting PDAC. Gemcitabine, which is the first-line therapy for PDAC, has been encapsulated in various NP formulations, including microbubbles (for ultrasound imaging) 191**,** luminescent photothermal NPs 192, and PLGA nanospheres containing fluorescent iron oxide NPs 193. Urokinase plasminogen activator receptor (uPAR)-targeted, PEGylated iron oxide NPs labeled with NIR dye (NIR 830-maleimide) and loaded with doxorubicin (DOX) or cisplatin were also evaluated in a syngeneic orthotopic model of PDAC. These NPs, when administered via the intraperitoneal route, enabled tumor visualization by NIR optical imaging and MRI and resulted in tumor growth inhibition 194. Similarly, human insulin-like growth factor receptor (IGF1)-targeted, NIR dye-labeled iron oxide NPs with DOX as therapeutic payload exhibited anti-tumor effects on orthotopic patient-derived xenografts (PDXs) and enabled NIR optical imaging and MRI 195. Additional examples of the recently published molecular imaging and theranostic nanoprobes for PDAC are shown in **Table 2**. Overall, NP-based imaging and theranostic agents have shown promise in preclinical studies.

### 3.3. Nanoscale delivery system for targeted therapy in PDAC

As discussed in Section 2, the pancreatic TME is a critical determinant of resistance to chemotherapy and immunotherapy. Nanocarriers have been designed to target tumor stroma by delivering inhibitors of signaling pathways involved in stromagenesis. In this regard, three secreted hedgehog proteins (Sonic, Indian, and Desert) and their downstream signaling molecules have been extensively studied and exploited to modulate tumor stroma 196, 197. Strategies employing nano-enabled siRNA and miRNA delivery systems targeting these pathways have been used in PC models 121, as detailed below. Efforts have also been directed to design NPs to exploit and/or modulate other pathophysiological or molecular hallmarks of PDAC, such as acidic pH, hypoxia, and stromal proteases.

#### 3.3.1. Stimuli-responsive NPs

Stimuli-responsive NPs take advantage of several unique PC features, including hypoxia, low tissue pH, and upregulated enzymes represented by cathepsins and matrix metallopeptidases, which are related to EMT. Gurka *et al.* designed a pH-responsive nanocarrier to co-deliver an extracellular signal-regulated kinase inhibitor and GEM 198. The triblock copolymer partially unfolds in response to the lower pH in the PC microenvironment, resulting in controlled release of payload and suppression of PC cell growth. Kulkarni *et al.* prepared hypoxia-responsive polymersome and lipid NPs for the targeted release of chemotherapeutics to PC cells 199, 200. In both studies, an azobenzene group was incorporated into the polymer that undergoes a reduction in response to elevated levels of reducing enzymes corresponding to hypoxia in the PC microenvironment. The hypoxia-responsive release of chemotherapeutics resulted in reduced cancer cell viability. In another study, a sequential release of GEM was realized using a dual enzymatic responsive nanocarrier 201. The PEG shield was first cleaved by the matrix metalloproteinase-9 overexpressed in the PC microenvironment and cathepsin-B upregulated in lysosomes.

#### 3.3.2. Antibody-mediated targeting

Tumor-specific antibodies can be incorporated into nanocarriers to target tumors in an antigen-specific manner and to promote site-specific accumulation. Antibodies targeting various upregulated receptors, including vascular endothelial growth factor (VEGF), epidermal growth factor receptor (EGFR), and carbohydrate antigens (e.g., CA19-9, CA125 Sialyl Tn), have been extensively tested for targeting nanomedicines to PC. McDaid *et al.* used the clinically approved anti-EGFR antibody (i.e., Cetuximab) for targeting PLGA NPs in order to lower off-target cytotoxicity and enhance drug efficacy in EGFR-resistant PC 202 The conjugation-induced targeting and apoptosis were demonstrated in several different cancer cell models, indicating a generalizable approach for nano-enabled enhanced drug efficacy. In another study, (1,2-diaminocyclohexane) platinum(II) (DACHPt)-based polymeric micelles loaded with OX were conjugated with an antigen-binding fragment of a novel tissue factor antibody 203. The antibody-conjugated micelles were rapidly internalized by PC cell line BxPC3 and localized in lysosomes and late endosomes. Further, a murine tumor model with subcutaneous BxPC3 xenografts was used to test the antitumor efficacy of DACHPt micelles. The antibody-conjugated DACHPt micelles exhibited superior tumor inhibition compared to non-targeted micelles and soluble drugs against established pancreatic tumors.

#### 3.3.3. Ligand-promoted targeting

In addition to antibodies, other biological molecules and ligand-targeted drug systems have been explored for cancer targeting 204. He *et al.* prepared a combination NP system with ECM-targeting aptamer, cell-penetrating peptide, and redox responsive release 18. Lin *et al.* conjugated an anti-EGFR peptide GE11 to a liposome nanocarrier to facilitate targeting specificity 205. The ligand targeting strategy was synergized with the co-delivery of HIF1α siRNA and GEM. The combined formulation enhanced drug uptake, increased apoptosis, and reduced tumor burden in a murine model. The aptamer GBI was released upon interaction with ECM component tenascin-C and exposed the cell-penetrating peptide for tumor cell internalization. The NP system was tested on PC spheroids and tumor-bearing nude mice, demonstrating improved drug efficacy and tumor regression. Lee *et al.* prepared polymer-coated magnetic iron oxide NPs conjugated with a urokinase plasminogen activator targeting peptide. This NP system realized the dual function of targeted GEM release and MRI contrast enhancement in a PC xenograft murine model 156.

## 4. Nanocarrier-driven immunomodulatory approaches for PDAC

Cancer emergence and progression often imply the failure of the immune system to detect tumor antigens and destroy malignant cells 206. Current vaccine approaches, which are based on protein, peptides, nucleic acids, or adoptive transfer of immune cells such as dendritic cells (DCs) or T cells, fail to achieve or stimulate the desired magnitude and/or the correct arm (i.e., phenotype) of the immune response to confer anti-tumor immunity with therapeutic benefits. While promising, these cell-based immunotherapies rely heavily on continual *in vitro* stimulation or cultivation of cells, which may induce immunological exhaustion, resulting in inadequate *ex vivo* expansion and/or shortened survival rate upon infusion, and ultimately low rates of successful clinical responses 207. The urgent demand to obtain precise control over the induction of desired arm(s) of the immune response has brought more attention towards the rational design of nanocarrier-based cancer vaccines (such as polymeric nanovaccines). These research efforts are based on a deep knowledge of how the immune system interacts with nanocarriers to generate strong and durable immune responses to effectively combat tumor cells 208, 209. The successful development of such nanocarrier-based cancer vaccines relies on addressing critical challenges, including (i) efficient delivery of tumor antigen(s) to antigen-presenting cells (APCs); (ii) suitability of vaccines to activate appropriate pathways within APCs and other immune cells; (iii) appropriate packaging and delivery of diverse vaccine components (antigens and immunological adjuvants) to generate optimal antigen-specific antitumor immune responses; and (iv) minimizing adverse reactions such as systemic inflammatory responses 210, 211.

### 4.1. Polymer chemistry and immune activation

Various natural and synthetic biodegradable and biocompatible polymers have been widely investigated and used to fabricate nano- and microparticles encapsulating single or multiple vaccine components. Most notably, the biodegradable and biocompatible copolymer PLGA has been extensively explored for controlled delivery of biologically active molecules (including vaccine constituents) 212. An important advantage of employing PLGA in vaccine delivery is its adaptability, suitability, and ease of manipulation of its chemical and physical properties, such as hydrophobicity/hydrophilicity, molecular mass, and crystallinity through changes in the monomer ratio, terminal group chemistry, size, and net charge 82, 90, 209. Thus, the physicochemical properties of PLGA-based particulate vaccines can be rationally optimized to allow targeted delivery of tumor antigens for the generation of antitumor immune responses. The terminal group characteristics make PLGA amenable to surface modifications for improved targeting 213. For example, a study performed with tumor lysate-targeted PLGA particles coated with biotinylated streptavidin stimulated stronger tumor-specific immune responses when compared to uncoated counterparts 214.

Polyanhydride particles have been reported to have an adjuvant effect in that they can stimulate DCs through binding to Toll-like receptors (TLRs) 83, 215. Another important characteristic of polyanhydrides is their tunable degradation rate and unique surface erosion mechanism dictated by copolymer composition 216-218. We have shown that varying the molar composition of polyanhydride copolymers can also have a significant effect on the properties of particles and, subsequently, the antitumor immune responses 218. One major factor is hydrophobicity, which plays a key role in the opsonization and cellular uptake of particles. For example, increasing the molar ratio of CPH in polyanhydride copolymer composition resulted in a significant increase in the hydrophobicity of particles and, in turn, stimulated more potent antitumor immune responses and improved their *in vivo* performance 218. Similarly, poly(phosphazenes), a class of biodegradable polymers, have been explored for their TLR stimulatory effects. Studies revealed that poly(phosphazenes) displayed strong avidity to soluble immune receptor proteins (e.g., mannose receptor) and certain TLR proteins 219, 220. Another example of a biodegradable polymeric biomaterial that has been recently investigated for vaccine delivery is poly(diaminosulfide) (PNSN) 221, 222. Particularly, the use of PNSN for cancer vaccines in a murine tumor model showed that mice vaccinated with tumor antigen-loaded PNSN particles had high levels of CTLs, and the formulation conferred protective immunity against the tumor challenge 223. Poly(beta-amino esters) have also been studied for their application as cancer vaccine vectors. These polymers have a unique branched architecture that provides a large chemical space for complexation and functionalization. Due to their cationic properties, poly(beta-amino esters) enhances cellular uptake and endosomal escape via the proton sponge effect 224, 225. Polymeric nanocarriers can provide effective solutions to these obstacles, and degradable polymers used for cancer vaccines are summarized in **Table 3**.

### 4.2. Mechanisms of immune induction by nanocarriers

**Figure 3:**
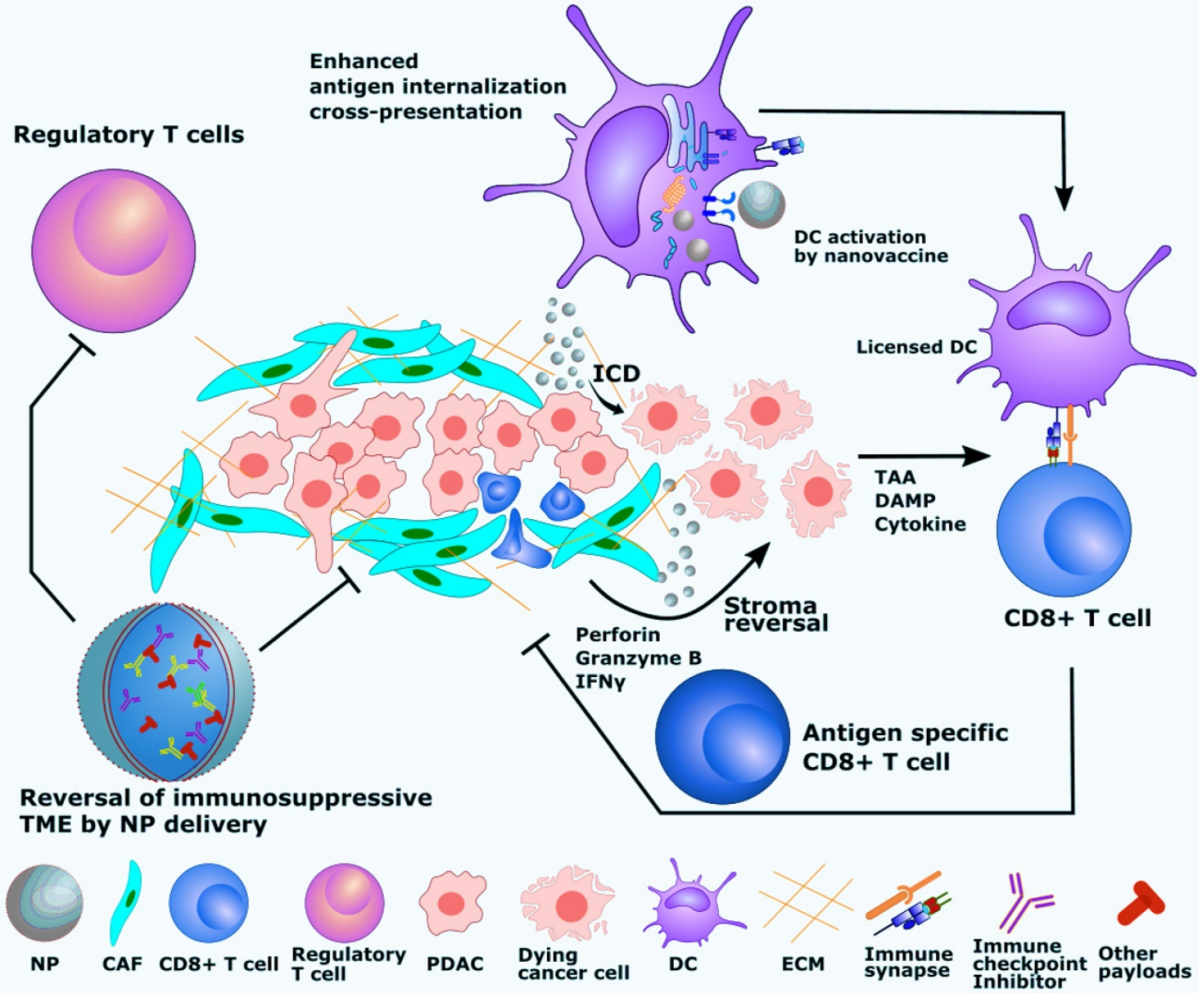
** Advantages of polymeric nanoadjuvants for PDAC immunotherapy.** Clockwise from the top, the figure shows how polymeric NPs: enhance exogenous antigen internalization by DCs, which can promote antigen transportation to secondary lymphoid organs and increase antigen persistence; improve antigen cross-presentation by increasing cytosolic delivery of encapsulated payloads in DCs, thus leading to more effective antigen-specific CD8+ T cell activation; enhance ICD and sensitize PDAC to immune cell recognition; induce higher levels of CD8+ T cell activation by licensed DCs or ICD based on *in situ* vaccination; enable more efficient removal of stroma, and enhance the reversal of immunosuppressive TME. NPs: Nanoparticles; ECM: Extracellular matrix; CAFs: Cancer-associated fibroblasts; DCs: Dendritic cells.

The mechanisms by which nanocarriers induce antitumor immune responses are dictated by how biomaterials interact with the host immune system. Interaction of nanocarriers with blood or interstitial fluid results in the rapid formation of a protein layer on the biomaterial surface, known as the “protein corona” 226. Nanocarrier surface chemistry, charge, and morphology have been shown to extensively impact immune activation, as reviewed elsewhere 227-229. In addition, the identity of NPs is redefined by the protein corona due to its impact on pattern recognition receptor (PRR) engagement, activation of the complement cascade, and cellular internalization. Following protein deposition on NP surfaces, leukocytes sense the biomaterial surface by surface receptors, which leads to downstream signaling events, including activation of inflammation-related transcription factors (e.g., NF-κB and NFAT) 230. These transcription factors further regulate a series of immune activation events such as cytokine and chemokine expression, which not only directly impact immune cell behavior, but also orchestrate global immune activation via modulation of vascular permeability and dilation. Another outcome of leukocyte interaction with biomaterials is the increase in oxidative stress because of enhanced mitochondrial activity (i.e., metabolic changes) and PRR-induced anti-microbial immunity 231. Recent studies also used the level of reactive oxygen species (ROS) as a measure of immune activation, which could be altered by biomaterial-based immunomodulation 232, 233. Among mononuclear cells, macrophages and DCs serve as the most effective APCs for T cell activation. In particular, DCs are the primary cell type responsible for cross-presentation and induction of antigen-specific CD8^+^ T cells. As one of the most heterogeneous cell populations, distinct T cell subsets can be identified by their activation status, antigen experience, and effector functions and play important roles in inducing optimal immune responses. Although T cells are rarely shown to adhere directly onto biomaterial surfaces and be activated hereby, their activation can be tuned by biomaterial-leukocyte interactions 234, 235. The multiple advantages provided by the physicochemical and mechanistic aspects of nanocarrier-mediated immunomodulation are summarized below and shown in **Figure 3**.

#### 4.2.1. Enhanced APC internalization

Nanocarriers with tumor-associated antigens (TAAs) are preferentially internalized by APCs, thus offering an increased magnitude of APC activation and dose sparing and leading to enhanced antigen processing and T cell activation 236, 237. One of the determining factors of the endocytic uptake pathway by APCs is the size of particles that deliver the cancer vaccine components. It has been found that nano-sized particles are readily internalized by pinocytosis, whereas micron-sized particles are taken up by the phagocytotic process 238. A study that compared the uptake of different sizes of antigen-loaded PLGA particles (0.3, 1, 7, and 17 µm) found that smaller particles were readily internalized by DCs, and this was associated with stronger stimulation of *in vivo* antigen-specific immune responses when tested in murine tumor model 90. Other studies have shown that nanoparticles less than 100 nm can potentially traffic on their own to the draining lymph nodes (DLNs), where they can be captured by the LN APCs, which may result in more efficient antigen cross-presentation and CTL priming 239, 240. In contrast, larger particles normally remain at the vaccination site and are phagocytosed by the migratory APCs, which then migrate to the closest DLN 239, 240. PDAC is often characterized with strong local immunosuppression and distant immunoremodeling 241, which renders ineffective antigen presentation by APCs and decreased co-stimulatory signaling to T cells. Systemic or intratumoral APC activation can be exploited to enhance T cell immunotherapy. Lorkowski *et al*. prepared lipid-based immune-stimulatory NPs (immune-NPs) for the co-activation of STING pathway and TLR4. The immune-NP is designed to target the tumor local innate immune cells and promote APC activation and proliferation. A high percentage of NP cellular uptake was observed in multiple organs and orthotopic Panc02 tumor concomitantly with increased tumor-infiltrating APCs 242, which is instrumental for T cell priming and recognition of cancer cells.

#### 4.2.2. Biomaterials with inherent adjuvanticity

Some biomaterial-based nanocarriers can provide immunostimulation, resembling conventional vaccine adjuvants. For example, NPs modified with hydroxyl and amino groups induced complement system-mediated immunostimulation 240, 243. Polyanhydride NPs also showed chemistry-dependent APC activation (e.g., elevated CD80/86 expression, cytokine secretion) 244. It has been suggested that such non-specific biomaterial-induced adjuvant effects could be attributed to a hydrophobicity-based danger-associated molecular pattern (DAMP)-like mechanism 245.

#### 4.2.3. Enhanced cross-presentation and induction of CTLs

Extracellular antigens need to be internalized and presented to major histocompatibility complex (MHC) class I molecules for effective induction of CTLs. Nanocarriers can enhance cytosolic delivery of TAAs, leading to endosomal escape and processing via proteasome into peptides loaded onto MHC I molecules for the induction of anti-tumor, antigen-specific CD8^+^ T cell immunity. Several endosomal escape mechanisms have been proposed 209. Among these mechanisms for nanocarrier-induced endosomal release is the “proton sponge hypothesis” 246. This strategy has already been demonstrated on PDAC models using polyethyleneimine modified aluminum hydroxide NP as a vaccine carrier to a Panc02-OVA tumor 247. The resulting nanovaccine induced antigen-specific immunity to Panc02 cells and regression of the established pancreatic tumor. Another study used liposome NPs to target mouse CD169^+^ DCs via ganglioside, a natural ligand of CD169. This NP was shown to increase antigen cross-presentation and target Axl+ DCs derived from PDAC patients 130. In addition to the reversal of PDAC TME, the use of targeted APC activation could be a powerful approach to further recruit CTLs.

#### 4.2.4. Lymph node delivery

Studies have shown that antigen accumulation at DLNs significantly enhances T cell activation 210. Conventional routes of vaccine administration induce suboptimal activation of CD8^+^ T cells due to insufficient antigen-loaded cDC1 migration to LNs 248. More recent studies with tumor models demonstrated that the co-delivery of adjuvant and antigen to LN is critical for optimal immune activation, making a strong case for NPs capable of loading multiple components 211, 212. A Japanese study analyzed LN metastasis in 429 PDAC patients and identified high incidence in advanced PDACs 249. Because of the limited therapeutic measures available to PDAC patients with DLN metastasis, immunomodulatory interventions to LN should get more attention. A case has been made by using PLA microspheres loaded with IL12 (IL12 MSs) to repolarize the pancreatic DLN immune profile in an orthotopic KCKO PDAC model 250. In this study, IL12 microspheres were tested in combination with stereotactic body radiotherapy (SBRT) and/or lymphatic ablation. IL12 microspheres + SBRT inhibited tumor growth and induced immune profile alteration, including expression of CXCL10, IFNγ, and granzyme B. Interestingly, the DLN excision partially abrogated these effects.

#### 4.2.5. Immunogenic cell death (ICD)

ICD is a specific type of cell death characterized by the release of DAMPs, inflammatory signaling molecules, and in the case of cancer cells, TAAs 251. ICD provides a combination of antigens, cytokines, and co-stimulatory molecules required for APC activation, and therefore can be instrumental for T cell priming. NPs loaded with cytotoxic reagents have been utilized for anti-tumor therapies by inducing ICD 58, 252. In addition to the tumoricidal effects, certain types of chemotherapeutics such as DOX and OX have also been reported to elicit ICD and thereby function as *in situ* vaccination against tumors 251, 253. A study that investigated the *in situ* immunization against both B cell (A20) and T cell (EL4) lymphoma tumor models with PLGA particles co-encapsulating DOX and CpG-ODN showed that the combination regimen was effective at generating systemic responses and reducing tumor burden, which was further enhanced by anti-OX40/anti-CTLA4 monoclonal antibody (mAb) therapies to improve T cell activation and overcome immunosuppression 254. Another recently reported example is the *in situ* immune stimulation against the B16.F10 melanoma tumor model with PEGylated PLGA NPs encapsulating DOX with or without anti-PD1 255. The median survival time of animals was extended to 55 days post-tumor challenge in comparison to 15 and 30 days for naïve and soluble DOX treated mice, respectively 255. Upon combining the DOX-loaded PEGylated PLGA NPs with anti-PD1 therapy, there was a synergistic effect, and the median survival time was not reached since 60% of mice remained tumor-free at the completion of the study 255. ICD-inducing nanoplatforms have also been tested in PDAC models. A supramolecular nanocarrier was used to co-deliver photosensitizer and prodrug in a Panc02 tumor model 256. The NPs were made from self-assembly of cyclodextrin-grafted hyaluronic acid, pyropheophorbide a (photosensitizer), and JQ1 (prodrug). The resulting NPs downregulated Panc02 tumor-associated immunosuppression and elicited ROS-driven ICD. By 40 days post-treatment, the multiple-component NP plus laser excitation significantly prolonged the survival of Panc02-bearing mice compared with control or monotherapy groups. Inhibition of tumor recurrence and metastasis was also observed up to the endpoint of the tumor study. Another study employed OX as the inducer of ICD in the Panc02 tumor model where OX was co-encapsulated with a siRNA against galectin-9/dectin-1 axis into bone marrow mesenchymal stem cell-derived exosomes 255. The combination therapy was shown to reverse the M2-like polarization of macrophages in the tumor and significantly inhibited orthotopic Panc02 tumor growth throughout the 28-day course study. More studies using nano-enabled mechanisms in PDAC models are summarized in **Table 4**.

### 4.3. Nanocarriers for PDAC immunotherapy

The approaches and concepts described in **Sections 4.1 and 4.2** have led to the design and study of nanocarrier-enabled PDAC immunotherapies. From the identification of novel TAAs to the preparation of nanoformulations to the characterization of immune activation and anti-tumor performance in various PDAC tumor models, researchers are moving forward to synergistic nano-driven immunotherapies for optimal anti-tumor efficacy. **Table 4** lists nanocarrier-enabled PDAC immunotherapies applied in distinct modalities (e.g., TME reversal, nanovaccine) that have been evaluated in various types of PC models.

### 4.4. PDAC tumor-associated antigens (TAAs)

Various TAAs have been investigated for both targeted delivery of therapeutic and diagnostic agents and for developing immunotherapy for PDAC. The majority of TAAs initially described for various cancers, including PC, are cell surface glycoproteins and cell surface receptors that are either aberrantly glycosylated and/or overexpressed and were initially used as biomarkers. Subsequently, several of these TAAs were exploited for payload delivery of therapeutic and imaging agents or direct targets for immunotherapy, antibodies, and small molecule drugs (**Table 2**). PDAC is characterized by overexpression of several mucins, high molecular weight glycoproteins that are either cell-surface tethered or secreted 257. Most cancer-associated mucins are encoded by multi-exon genes and characterized by variable number tandem repeat (VNTR) domains that are heavily O-glycosylated and secreted. By virtue of their overexpression, extensive splicing, mutations, and aberrant glycosylation in cancer, carcinoma mucins are promising neoantigens, while the presence of repetitive VNTR epitopes makes them excellent targets for payload delivery 257, 258. Consequently, several membrane-tethered (MUC1, MUC4, and MUC16) and secretory (MUC5AC) mucins have been explored as immunogens that were delivered using nanocarriers and for the development of immunotherapies in PDAC 258-262. Several other cell surface glycoproteins (carcinoembryonic antigen-CEA), mucin-associated carbohydrate epitopes (Sialyl T, Sialyl Lewis^a^), and mucin-interacting proteins (mesothelin, galectins) have also been investigated for PDAC immunotherapy 263-267. Similarly, antibodies and ligands of several growth factor receptors (EGFR, HER-2, and VEGFR) have been used for the delivery of therapeutic and diagnostic payloads using nanocarriers in PDAC 202, 268-272. In addition to cell surface TAAs, tumor-specific intracellular targets (K-Ras^G12D^, telomerase) have been explored for the immunotherapy of PDAC. Various PDAC TAAs that have been explored for payload delivery are summarized in **Table 2**, while preclinical and clinical studies investigating their utility for immunotherapy are reviewed elsewhere 10, 273-275.

Due to the uniquely immunosuppressive TME associated with PDAC, immunomodulation strategies have focused on normalizing the desmoplastic stroma and restoring immune cell function and infiltration (**Table 4**). A promising nanoscale strategy was described by two studies from the Huang group 62, 276. Plasmid genes encoding IL10, CXCL12, or PDL1 protein traps were encapsulated and delivered by liposome-protamine-based NPs for transfection. The delivery of high-affinity trap protein was designed to compete with the binding of the cytokines to their cognate receptors to interfere with factors that contribute to the immunosuppressive TME. In both studies, the NPs were shown to accumulate preferentially at the site of the tumor and induce downregulation of immunosuppressive response in an orthotopic tumor model of developed by implantation of KPC cells 62, 276. Nanoscale formulations have also been exploited for targeting the delivery of TAAs as nanovaccines. Liposome-based nanocarriers represent the most well-studied platform in human cancer vaccine clinical trials 277, 278. A liposome carrier was conjugated with ganglioside, a CD169 ligand, to facilitate the targeting and delivery of PDAC antigen WT1 to CD169^+^ APCs in patients. The resulting liposomal NP simultaneously delivered TLR4 ligand monophosphoryl lipid A (MPLA) and WT1 antigen to various types of DCs and was shown to induce the *in vitro* expression of IFNγ from a T cell line 130. Another study used PDAC mesothelin (MSLN) antigen-containing virus-like particles (VLPs). VLPs are considered effective carriers for immunotherapy due to their intrinsic ability for immunostimulation. Therapeutic vaccination with VLPs containing MSLN induced CTL responses and limited the expansion of Treg cells following orthotopic implantation of Panc02 tumor cells, resulting in inhibition of tumor progression 279.

### 4.5. Combination nano-platforms for CTL induction and tumor control

A key advantage of nanoscale delivery systems is their ability to combine multiple components/payloads. Nanocarriers can optimize the delivery of diverse payloads, coordinate co-delivery of multiple payloads, enhance their synergistic effects, and provide immunostimulation. For example, LCP NPs were loaded with 5'triphosphate double-stranded BCL2 siRNA and conjugated to aminoethyl anisamide (AEAA) 60. LCP NPs are ideally suited for ppp-dsDNA delivery because the phosphate-rich payload is easily loaded into the phosphate-rich particles 280. AEAA is the ligand for the sigma-2 receptor that is upregulated in many PCs 281. Its conjugation to the NP improved localization into tumor cells and tumor-adjacent fibroblasts in a KPC mouse model. This is the ideal location for ppp-BCL2 dsRNA delivery because it simultaneously acts as a RIG-I agonist inducing pro-CTL Th1-skewing (e.g., IFNα/β) cytokines and silences the anti-apoptotic BCL2 gene, making the tumor more vulnerable to the immune response.

Another nano-system combines the activity of the small molecule IDO1 inhibitor indoximod (IND) with OX-loaded NPs 58. IND can help reverse the immunosuppressive TME by maintaining local tryptophan levels 282, but the drug has poor retention in the TME when delivered orally 283. To overcome this, a phospholipid group was added to IND, creating a prodrug that self assembles into spherical nanovesicles in an aqueous solution. This lipid bilayer was stabilized with MSNs, which are ideal for the release of loaded OX, an inducer of ICD associated with KPC cells 58. This approach not only improved the pharmacokinetic stability and tumor penetrance of both drugs, but co-delivery produced a synergistic improvement in the TME CD8^+^/Foxp3^+^ ratio, DC intercalation, and tumor control in a KPC mouse model.

### 4.6. Overcoming immune exclusion of CTLs

While the generation of tumor-specific CTLs is an important step in anti-cancer immunotherapy, the effectiveness of the immune response is limited by access to the tumor itself. As mentioned in Section 2, a hallmark of PDAC is the presence of extremely desmoplastic stroma, making most pancreatic cancers immune-excluding tumors or “cold tumors” and shielding them from CTL activity. However, depletion of the stroma alone has proven detrimental in some PDAC models, allowing the escape of a less differentiated, more aggressive tumor phenotype and the infiltration of undesirable regulatory B and T cells 33, 284. One strategy to address this issue is to improve CTL infiltration without disrupting the fibrous component of the desmoplastic stroma by normalizing intratumoral vasculature. To this end, cyclopamine (CPA), an SHH pathway inhibitor, and PTX, a chemotherapeutic agent, were encapsulated into biodegradable polymeric micelles 285. SHH activates CAFs and contributes to the development of desmoplastic stroma, but excessive ablation leads to increased metastasis 33. However, small doses of CPA delivered by the micelles increased intratumoral vascularization without reducing collagen content and further controlled tumor growth with localized PTX delivery. This led to increased CTL infiltration, slowed tumor progression, and increased sensitivity to anti-PD-1 in murine PDAC models without the systemic toxicity associated with PTX 53, 285.

Another approach is to disrupt the fibrous tissue and tumor growth simultaneously to prevent increased metastasis. This has been accomplished in an animal model using a cholesterol-modified CXCR4 antagonist (PCX) that self-assembles into NPs. These particles have been shown to limit tumor invasiveness by blocking CXCL12/CXCR4 signaling and can be simultaneously used as a vector for transfection 286. In one study, PCX NPs were used to transfect tumor cells with a siRNA against NCOA3, a key regulator of PDAC pathology (e.g., it regulates mucin, enhances inflammation, and promotes tumor growth) 287. In another study, PCX was used to encapsulate two RNA therapeutics: anti-miR-210 and siKRAS^G12D^ 288. miR-210 is a hypoxia-induced miRNA important in the induction and activity of activated pancreatic stellate cells (PSCs) 289, while KRAS mutations are central drivers of most PDACs 290, 291. In both studies, PCX NPs increased perfusion of the tumor without detrimental effects, reducing both primary tumor size and metastatic events.

While some models of stroma-only targeting have reduced PDAC survival, a multi-faceted “nano-sapper” strategy utilizing a CaP liposome carrier has been shown to co-deliver phosphor-alpha-mangostin (PM), a prodrug that reduces liver fibrosis 292, and the pleiotropic inflammatory cytokine LIGHT 293, 294 delivered via plasmid vector 67. The CaP nanocarrier was decorated with an FHK peptide to target the liposome against tenascin-c expressing PSC surrounding the tumor 295, 296. While this regimen did not target tumor cells directly, it effectively attenuated the physical barrier to allow increased CD4^+^ and CD8^+^ T cell infiltration. This treatment also reduced overall tumor infiltration of regulatory immune cells and inhibited tumor progression in murine PDAC models.

Other approaches in PC immunotherapy have focused on SLNs, and hybrid MSNs. SLNs facilitate a more precise release of the immune reagents, mitigate off-target CTL responses, and effectively harness the humoral and cellular immune responses against cancer cells 134. Stimuvax (i.e., MUC1-specific), Tecemotide (i.e., MUC1-specific), and sHER2+AS15 are notable examples of liposome-based cancer nanovaccines that have progressed through phase-II/III clinical trials to treat melanoma and NSCLC, breast cancer, and PDAC, respectively. Recently, various immunomodulator or agonist-loaded biodegradable MSNs have been studied for cancer immunotherapy 297, 298. In one study, MSNs entrapping Ca/Mg/Zn was used as a biodegradable adjuvant to stimulate Th1-skwed immune responses to ovalbumin (OVA) and protect against E.G7-OVA lymphoma 298. Researchers have also used PD-1-based tumor targeting in concordance with inhibition of TGF-β pathways and showed improved survival in cancer-bearing mice 299. Additionally, using a pilot *ex vivo* study, T cells were loaded with SPION in order to facilitate T cell accumulation in the tumor using an external magnetic field 296.

### 4.7. Nanoparticulate systems to promote immune checkpoint therapy for PDAC

Despite the growing number of PDAC clinical trials involving immune checkpoint inhibitors (ICI) 300, pembrolizumab is the only FDA-approved anti-PD1 inhibitor for a fraction of PDAC patients with repair-deficient mismatch and instability-high microsatellite 301. The application of ICI was hindered by the non-inflamed nature of the pancreatic tumor and therefore resulted in a low frequency of existing tumor-specific T cells, which was further complicated by the immunosuppressive TME. ICI can also require repeated and high doses, which can lead to immunotoxicity 302 and other adverse events 303. Even combination with chemotherapy or other types of immunotherapies provided limited improvement. A recently completed phase II trial using GVAX and Listeria-based vaccine reported efficacy with nivolumab (anti-PD1) on patients with metastatic PDAC 304. The nivolumab (3 mg/kg) was administrated intravenously every 3 weeks for 6 cycles. Albeit promising CD8^+^ T cell increases and reduced immunosuppression in patients, the use of nivolumab did not increase overall survival. A higher-grade adverse event rate (≥ 3) was also reported, emphasizing opportunities to optimize ICI delivery and manage immune activation-related toxicity. In this regard, the use of nanocarriers could be a useful approach to enhance ICI. Many efforts have focused on targeting specific cell subsets 305, 306, normalizing TME 307, and increasing tumor immunogenicity 308, 309. More recently, several studies have described nanocarrier-enhanced ICI immunotherapy against PDAC models in mice 256, 310, 311. In addition, Yu *et al.* demonstrated a comprehensive stimuli-responsive NP system to induce hyperthermia with the goal of overcoming tumor barriers and promoting ICI 311. The anti-PD-L1 molecule was released from the dual-responsive liposome NPs to overexpressed fibroblast activation protein and irradiation. The NPs were shown to accumulate at the orthotopic tumor, increase T cell tumor infiltration, and reduce both primary and metastatic tumors. Additional nano-abled approaches are being studied for promoting ICI in PDAC.

## 5. Perspectives and outlook

Despite decades of progress in our understanding, PDAC remains one of the most lethal and challenging human malignancies to treat. Although more basic research and clinical studies are needed to develop potent treatments for PDAC, the advent of nanocarrier-based treatments holds immense promise to enhance patient survival rates. Some areas of future research in this area and their associated challenges are provided below.

The pancreatic TME is one of the critical drivers of therapy resistance. The obstructive stroma not only impedes the delivery of chemotherapeutic agents, but in addition various secretory components, particularly the ECM, proteases, and cytokines also orchestrate the development of an immunosuppressive milieu. While stromal targeting was envisioned to revolutionize the landscape of PC therapy, the early enthusiasm was tempered by the failure of clinical trials targeting pro-fibrogenic pathways, particularly SHH using pharmacological agents 295, 312, 313 and enzymatic degradation of ECM using PEGylated hyaluronidase [Pegvorhyaluronidase alfa (PEGPH20)] 314, 315. However, the focus now has shifted towards stromal modulation rather than depletion (unlike SHH inhibitors and hyaluronidase, which can also lead to altered tumor immune microenvironments). In fact, several anti-stromal therapies have demonstrated an altered immune landscape in tumors, including enhanced T cell infiltration and changes in macrophage polarization, thereby sensitizing the tumor to immune checkpoint blockade agents 316. Several clinical trials are now examining anti-stromal agents in combination with immune checkpoint blockade agents. Nanomedicine is providing some answers, such as NP-encapsulated SHH inhibitors that provided stromal modulation 285. Similarly, Losartan, an angiotensin II receptor antagonist, has shown promise in vascular and stromal remodeling and has been demonstrated to enhance the delivery and efficacy of chemotherapy 317, 318. Variable performance of anti-stromal therapies can be attributed to the heterogeneity and plasticity of stromal cells, particularly CAFs, whose diverse origins and phenotypes are only beginning to be understood. While NP-based therapies are believed to accumulate in the sleeves of the tumor vasculature due to the EPR effect, the dense stroma limits their delivery. Thus, evaluating NPs in conjunction with stromal modulators like Losartan and other agents that enhance perfusion can potentially improve intratumoral delivery.

Advancements in polymer design and chemistry have culminated in the synthesis of versatile polymeric delivery systems for encapsulation of TAAs and immune adjuvants. Specifically, controlled reversible-deactivation radical polymerization mechanisms such as reversible addition-fragmentation chain transfer (RAFT) polymerization and atom transfer radical polymerization have resulted in the production of well-characterized polymers and facilitated the polymerization of multifunctional monomers 319. For example, RAFT polymerization has been exploited to synthesize versatile amphiphilic copolymers comprising a polycation-rich polymer (dimethyl aminoethyl methacrylate for CpG ODN complexation) with a pyridyl disulfide functional group (for antigen conjugation) and a hydrophobic endosome-lytic component (for endosomal escape), which form self-assembled micelles and enhance antigen cross-presentation by promoting cytosolic delivery. These micelles enable dual delivery of both tumor antigen and immunostimulatory CpG ODN 320. Additionally, RAFT polymerization has been employed to synthesize mannose- and acetylglucosamine-containing glycopolymer delivery systems capable of targeting C-type lectin receptors, which are expressed on APCs such as DCs and macrophages 321, 322. Such molecular targeting capabilities would confer an additional level of targeting specificity toward the rational formulation of PDAC immunotherapies.

Nano-enabled strategies can also improve PDAC sensitization to chemotherapy by combining the delivery of chemotherapeutics agents, small molecule drugs, and gene therapy. However, despite this diversity, these strategies rely upon the induction of the patient's natural anti-tumor immune response. While there has been some success with this approach, a potential avenue for improvement is the concomitant induction of a PDAC-specific CTL response. In this regard, combining targeted nano-driven immunotherapy with PDAC stromal penetration and accumulation of chemotherapeutic agents within tumors could be beneficial. The success of such approaches is predicated upon tailoring them for PC patients via genetic screening and characterizing tumors in clinical trials to better understand tumor resistance to chemotherapeutic drugs. Combining nanocarrier treatments or immunotherapies with ICI is a highly promising approach for PDAC that can abrogate the immunosuppressive TME, enhance tumor immunogenicity, and extend patient survival times. We anticipate an increase in the number of clinical studies that will pursue such strategies.

The development of multifunctional NPs with diverse and complementary functionality can be used to enhance the efficacy of PDAC treatments. Multifunctional NPs can accommodate synergies in terms of co-delivery of diverse payloads (e.g., therapeutics, immune-stimulatory molecules, and TAAs), targeting capabilities, and theranostic functionality. If successful, these multifunctional NPs can provide dose-sparing, reduce cost, and lower the toxicity of PDAC therapeutics. Such versatile approaches can also be used to treat metastasized tumors leading to a “systems approach” that anticipates adverse events and broadens therapy options.

Therapeutic strategies using EVs and exosomes have progressed rapidly in recent years 323. Exosomes are being envisioned as an alternative natural delivery system for targeted therapeutics. Additionally, the introduction of exosome delivery, as well as molecular and nanotechnological advances in precision medicine, is enabling the scientific community to develop improved treatment options 324. This is exemplified by recent efforts to utilize reassembled pancreatic tumor cell-derived exosomes for delivering photosensitizer to tumors for photoacoustic imaging-guided photodynamic and immunotherapy 325. Despite these advancements, several challenges exist with respect to the large-scale production and purification of EVs and particularly exosomes. While MSC-derived exosomes have been used in preclinical and clinical studies, their interactions with the components of the immune system remain poorly understood. Other hurdles include the need for a gold-standard method to isolate and precisely identify exosomes and an ideal low-cost strategy with high reproducibility and efficient exosome purification. Furthermore, most exosome engineering applications targeting PDAC treatment are largely limited to pre-clinical studies. Advancing research to overcome these shortcomings could pave the way to novel therapies in PDAC and other cancers.

Nanocarrier-based approaches have been predominantly employed to deliver miRNA, siRNA, and plasmids for gene silencing and expression. These approaches have their own limitations. While the gene-silencing effects of siRNA are short-lived *in vivo*, miRNAs regulate multiple targets that can lead to off-target effects. CRISPR-Cas9 based approaches have emerged as highly selective and effective gene-expression manipulation platforms. Recently nanocarrier-based delivery of CRISPR/Cas9 system has been used for manipulation of tumor microenvironment for modulation of response to immunotherapy in conjunction with chemotherapy and photodynamic therapy in melanoma 326, 327. It will be of interest to evaluate similar systems for the modulation of pancreatic TME and augment response to chemotherapy and immunotherapy.

PDAC is immunologically cold due to the lack of neoantigens and other immunosuppressive mechanisms that operate in the TME. Recently, it has emerged that it is not the neoantigen quantity but quality that dictates the effectiveness of immune response and patient outcome 262. The advent of personalized medicine and advances in computational and data analytics has encouraged greater levels of genomic, transcriptomic, and epigenomic profiling, TCR sequencing, and neoantigen profiling in conjunction with mutational loads. Integration of such data emanating from preclinical and clinical studies can help identify meaningful antigens that may not be abundant but are effective, and the nature of immune responses likely to be elicited by these antigens can be predicted. Modeling these aspects in preclinical studies can pay rich dividends not only in developing effective vaccines but also in monitoring/characterizing immune responses more dynamically in clinical trials. Informatics methods can also help determine the compatibility of a given neoantigen with nanocarrier platforms, leading to rational design approaches.

The field of nanocarrier-based therapies has dramatically expanded over the past several years. However, only a few anticancer nanomedicines, including drug-antibody conjugates, have thus far made it to the clinic. In total, there are nearly 250 clinical trials in the United States evaluating the potential clinical benefit of NP-based formulations. Most of these clinical studies are performed as combination therapies, while only a few studies are focused on the use of NP-based formulations as monotherapies. Advancing NP-based delivery systems from the laboratory bench to the bedside requires addressing challenges. One of these challenges is that results generated from *in vivo* efficacy and safety studies typically performed in animal models do not necessarily reflect equivalent outcomes in humans since the *in vivo* fate of a tested nanomedicine or nanovaccine and its interaction with blood components can be highly variable 328, 329. Another limitation is the difficulty that can be encountered in attempting to scale up the production of complex nanocarrier systems, which would be necessary for translation to the clinic. A further challenge that may impact the progress of nanomedicines to the clinic is that there are few contract facilities that have the capacity to reproducibly synthesize complex nanomedicines under cGMP conditions for use in clinical trials. The traditional approach of developing and exploring new anti-cancer nanomedicines involves varying certain parameters such as size and surface charge or chemistry. New strategies such as microfluidics or nanofluidics can help generate large libraries, which can facilitate the systematic screening of multiple parameters to maximize opportunities for the rational design of anti-cancer nano-formulations. Such approaches can enhance the success rate in terms of clinical performance and high-impact care for PC patients.

## Figures and Tables

**Table 1:** Representative examples of FDA-approved or clinical-stage nanomedicines for PDAC therapy

Nanomedicine	Nanocarrier	Payload/coating	Cancer type	Advantages	Approval	Ref.
Abraxane^®^ ABI-007	Albumin	paclitaxel	PDAC	Increased site-specific delivery, Improved solubility	FDA	330
Lipotecan^®^	PEG-PGA micelle	TCL388 HCl	PDAC	Better therapeutic effect, Prolong circulation, Low toxicity	FDA	331
Genexol-PM^®^	mPEG-PLA micelle	Paclitaxel	Metastatic PDAC	Improved solubility/efficacy, Reduced toxicity	FDA, Korea	332, 333
Doxil^®^	Liposomal	doxorubicin	PDAC	Increase site-specific delivery, Decrease systematic toxicity	FDA/Phase I/II	334
Onivyde^®^	PEGylated Liposome	Irinotecan	PDAC	Increased delivery to a tumor site, Low systematic toxicity	FDA	335
Lipoplatin^®^	Liposome	Cis-platin	PDAC	Specific delivery, Reduced toxicity	Phase II/III	336
EndoTAG^®^ -1	Liposome	Gemcitabine	Locally advanced & metastatic PDAC	Provide great potential and better treatment options than Gemcitabine alone	Phase III	337
MSC-derived exosomes	Exosome	KRAS G12D siRNA	Metastatic PDAC	Direct specific targeting, Improved therapeutic efficacy	Phase II	338

**Table 2:** Tumor-associated antigens (TAA) investigated for the delivery of therapeutic payloads in PDAC

TAA	Nanoparticulate carrier	Surface modifier/encapsulation of	Therapeutic/imaging Cargo	Application	Phase of Investigation	Modality	Ref.
MUC1	PLGA	MUC1 Ab (TAB004)	Paclitaxel	Ab-mediated Drug Delivery	*In vivo*	Therapy	88
	Iron oxide	MUC-1 peptide (EPPT)	Gemcitabine/Cy 5.5 dye	MRI/Drug delivery	*In vivo*	Therapy	339
MUC4	CPG & CPTEG	MUC4β protein	MUC4β	Immunotherapy	*In vitro*	Therapy	81
	MnMEIO-silane-NH2-mPEG	Anti-MUC4 Ab	MnMEIO	MRI	*In vitro* & *in vivo*	Imaging	340
MUC5AC	Liposome	RA-96 Fab	Indocyanine green (ICG)	Tumor imaging	*In vivo*	Imaging	186
CEA	Lipid-polymer	CEA Ab	Paclitaxel	Drug Delivery	*In vitro*	Therapy	264
CA19-9	mPEG-PLGA-PLL	CA19-9 Ab	Paclitaxel	Drug delivery	*In vitro*	Therapy	267
	Liposome	CA19-9 Ab	Doxorubicin	Ab-mediated drug delivery	*In vitro* & *in vivo*	Therapy	263
KRAS G12D	Glycol-Poly-L-lysine copolymer	Human scFv (CD44v6) Ab	siRNA	siRNA delivery (Gene therapy)	*In vivo*	Therapy	341
VEGF	PEG-CCP block copolymer	siRNA	siRNA	mRNA knockdown	*In vitro*	Therapy	270
	Graphene oxide	siRNA	siRNA & Doxorubicin	Combination therapy	*In vivo*	Therapy	342
Mesothelin	Iron oxide@SiO_2_	Anti-mesothelin Ab	IONPs	MRI	*In vitro*	Imaging	265
EGFR	CPT-PLGA	Cetuximab	Camptothecin	Antibody-mediated drug delivery	*In vitro*	Therapy	202
	BSA	Erlotinib	Parvifloron D	Targeting of EGFR	*In vitro*	Therapy	271
	Magnetic albumin	Cetuximab	Gemcitabine	MRI/Drug delivery	*In vitro*	Theranostic	343
	Silica NPs	Cetuximab	ZnPcOBP (Zinc Phthalocyanine)	PDT/PTT	*In vitro*	Therapy	344
	Liposomal formulation	EGFR (Cet) Ab	Benzoporphyrin derivative	*In vivo* photoacoustic imaging, PDT/PTT	*in vitro* &* in vivo*	Therapy/ imaging	345
HER2	Chitosan	HER-2Ab	Gemcitabine	Drug delivery	*In vitro*	Therapy	268
	Iron oxide	HER-2 Ab	Gemcitabine	MRI/Drug delivery	*In vivo*	Theranostic	193
Retinoic acid	Gold	Retinoic acid	siRNA	TME modulation & HSP47 targeting	*In vitro* & *in vivo*	Therapy	21
	Iron oxide	Retinoic acid	Gemcitabine	TME modulation	*In vitro*	Therapy	346
	PEG	PEG-Retinoic acid (PGRA)	Gemcitabine	TME modulation	*In vitro*	Therapy	347
CA19-9	Liposomes	CA19-19 diabody	^124^I	Emission tomography	*In vivo*	Imaging	348
	Carbon QDs	CA19-9 Ab	QDs	Fluorescence	*Ex vivo*	Imaging	349
	Gold	5B1 Ab	^89^Zr	PET	*In vivo*	Imaging	189
CD44	Iron oxide	CD44 Ab	Hyaluronic acid	MRI	*In vivo*	Imaging	350
uPAR	Iron oxide	ATF peptide	Gemcitabine	MRI//drug delivery	*In vivo*	Theranostic	156
Shh	Iron oxide	Shh (5E1) Ab	Cyclopamine	MRI//drug delivery	*In vivo*	Theranostic	351
Plectin-1	Iron oxide	Plectin-1 peptide	IONPs	MRI	*In vivo*	Imaging	352
	Iron oxide	Plectin-1 Ab	Cy7 dye	MRI/Fluorescence	*In vitro* & *in vivo*	Imaging	353
IGF-1	Iron oxide	IGF-1 Ab	Doxorubicin	MRI/Drug delivery	*In vivo*	Theranostic	195
Galectin-1	Iron oxide	t-PA-ligand	IONPs	MRI	*In vivo*	Imaging	354
	Iron oxide	Galectin-1 Ab	IONPs	MTAI	*In vivo*	Imaging	355
Glypican-1	Gold	Hyaluronic acid	Oridonin	NIRF/MRI/Drug delivery	*In vivo*	Theranostic	356
Neuropilin	Hsp 16.5 nanocages	iRGD peptide	Gadolinium	MRI	*In vivo*	Imaging	357
CEA CA19-9	mPEG-PLGA	CEA & CA19-9 Ab	Paclitaxel	Ab mediated drug delivery	*In vitro*	Therapy	266
EGFR, STAT3	PLGA	EGFR, STAT3 Ab	Alantolactone & Erlotinib	Dual targeting of EGFR & STAT3	*In vitro*	Therapy	269
MUC4, CEA, CD44	Iron oxide-PEG	MUC4, CEA & CA19-9	Paclitaxel	US/Drug delivery	*In vivo*	Theranostic	358
Cathepsin E (CTSE)	AuNPs	U11 peptides, 5-ALA (CTSE-sensitive prodrug), Cy5.5 dye	5-ALA and fluorescent dye Cy5.5	Optical imaging, PDT/PTT	*In vivo* &* ex vivo*	Therapy/ imaging	359

**Table 3:** Degradable synthetic biomaterials used in vaccine platforms

Polymer	Chemical Formula	Properties/Functions	Ref.
Poly(lactide-*co*-glycolide)	[C_3_H_4_O_2_]_x_[C_2_H_2_O_2_]_y_	Can be targeted to antigen-presenting cells, and their particulate nature can increase uptake and cross-presentation	90, 214
Polyanhydride	[CO-R-CO_2_]_n_	Surface erosion (tunable release rates) and inherent adjuvant properties	83, 215, 217, 218
Poly(phosphazene)	[N=PR_1_R_2_]_n_	Water-soluble and function as adjuvants	219, 220
Poly(diaminosulfide)	[R-N-S-N-R]_n_	Highly stable in neutral aqueous solutions while at lower pH conditions, the N-S-N linkage degrades faster, generating accelerated release kinetics	221-223
Poly(beta-amino ester)	[R_2_N-RCO_2_R]_n_	Readily phagocytosed and promotes *in situ* expression of chimeric antigen receptor genes	224, 225

**Table 4:** Nanoscale immunotherapy studies related to PDAC

Nano-enabled mechanism	Nanomaterial composition	Main results	Tumor model	Ref.
**Reversal of immunosuppressive TME**				
Enhanced cellular uptake and tumor penetration	mPEG-PEI-coated AuNP loaded with ATRA and siHSP47 for stromal modulation	Reversal of activated pancreatic stellate cell; ECM reduction Improved chemotherapy	PANC-1/pancreatic stellate cell co-inoculated subcutaneous xenografts	21
Nanocarrier enhanced co-delivery and drug efficacy	Self-assembled nanovesicles or lipid bilayer coated mesoporous silica NPs encapsulating inhibitor for immunosuppressive IDO pathway	Induced immunity against subcutaneously injected and orthotopic tumor challenge Increased CTLs, Decreased Tregs	Orthotopic pancreatic implant KPC model	58
Enhanced biodistribution and tumor accumulation	Liposome-protamine-DNA NP encapsulating plasmid encoding CXCL12 and IL10 trap	Activation of various suppressed immune cells in TME	Orthotopic, KPC PC, and 4T1 triple-negative breast cancer models	61
Reduced toxicity, enhanced transfection, and ECM targeting	Calcium phosphate core with thin-film from cholesterol, DOTAP, and PEG conjugated with ECM targeting FHK peptide	Successful transfection, Increased CTL tumor infiltration, Tumor site accumulation, Tumor site accumulation vascular normalization	Orthotopic Panc02 and KPC cell line derived pancreatic tumors	67
Exosome enhanced endocytosis via anchor protein	Exosomes derived from mesenchymal cells carrying siRNA for KRAS	Exosome enabled superior antitumor performance in various *in vitro and in vivo* cancer models	PANC-1 orthotopic xenograft tumor; KTC and KPC genetically engineered mouse PDAC models	142
Improved pharmacokinetics and toxicity	Liposome-protamine-DNA NP encapsulating plasmid encoding CXCL12 and PD-L1 trap	Improved antitumor response against KPC, allografts, and suppressed metastases;Enhanced T cell infiltration	Orthotopic pancreatic implant KPC allograft	276
Exosome accumulation at the tumor and enhanced payload efficacy	Exosomes derived from mesenchymal cells co-loaded with siRNA and OX	Accumulation of exosomes at the tumor site; Exosome-enhanced downregulation of immunosuppression and ICD; Improved profile of tumor-infiltrating immune cells	Orthotopic Panc02 syngeneic PDAC tumor model	360
Micelle pH-sensitive co-delivery of GEM	GEM and paclitaxel codelivery micelles based on a polyethylene glycol-polyarginine-polylysine (PEG-pArg-pLys) platform	Improved chemotherapy and immune cell infiltration; Stroma disruption; Decreased metastasis	MiaPaCa-2 tumor orthotopic PDAC xenograft model	361
**PDAC nanovaccines**				
Conjugated ligand enhanced internalization Enhanced cross-presentation	Ganglioside-liposome (EPC/EPG/cholesterol-based liposomes) nanovaccine loaded with WT1 or gp100 antigen targeting CD169	CD169 dependent liposome internalization by model DC Activated antigen-specific T cell line Activated patient-derived DCs	Samples derived from PDAC or melanoma human patients	130
Viral protein-induced immune stimulation	Insect cell produced MSLN antigen containing VLPs	Activation of MSLN specific CTL Decrease of tumor-infiltrating Tregs Therapeutic vaccine-induced tumor inhibition	Orthotopic PDAC syngeneic Panc02 pancreatic cancer mouse model	279
ICD TME targeting	Nanoparticulated mushroom Schizophyllan complexed with a humanized TLR9 agonistic CpG DNA	Proved need for innate immune component IL12p40 and type I interferon Phagocyte targeting in TME Tumor site accumulation	PC peritoneal dissemination model	362
Cationic liposome enhanced CpG delivery; Enhanced cytosolic delivery	Peptide-CpG-DNA -liposome lipoplex vaccine encapsulating TM4SF5 antigen	Antibody-mediated cancer cell inhibition Prophylactic tumor prevention	Transfected Panc02 human TM4SF5 expressing cancer model	363
Micelle enhanced stability and gene delivery	PEG catiomer and DNA polyplex micelles encapsulating gene encoding SART3 antigen, adjuvant CD40L, and GM-CSF	Observed cytotoxicity and proliferation for splenic CTL and NK cells; Therapeutic vaccination against various tumors; Analysis by CD4/CD8 T cell depletion assay	Various cancer cell line and tumor model	364
Enhanced antigen delivery, cytosolic delivery, and cross-presentation	Polyethyleneimine modified aluminum hydroxide NPs	*In vitro* DC activation and cross-presentation assay Vaccine-induced activation and proliferation of IFN-γ expressing CTL; Inhibition of established Panc02 tumor	Panc02 subcutaneous syngeneic pancreatic tumor model	247
**Other nanoscale immunotherapeutic strategies**				
Enhanced biodistribution and prolonged delivery	Lipid calcium phosphate NPs encapsulating dsRNA	Induction of Th1 response Increased CTL activation over Treg Analysis by CD4/CD8 T cell depletion assay Inhibition of established pancreatic tumors	Orthotopic KPC allograft PC tumor model and subcutaneous allograft BPD6 melanoma tumor	60
Enhanced NP biodistribution and cellular uptake	Lipid, cholesterol, and PEG-based NPs encapsulating STING and TLR4 agonist	Increase of tumor-infiltrating immune cells Inhibition of established subcutaneous Panc02 tumor	Orthotopic and subcutaneous Panc02 syngeneic pancreatic tumor model	242
LN delivery and prolonged release	PLA microspheres loaded with IL12	Intratumoral injection of IL12-MSs altered DLN cytokine profile; IL12-MS plus SBRT efficacy was reduced by DLN ablation	Orthotopic KCKO tumor model	250
Co-loading by self-assembled NP and tumor-targeting	Supramolecular NP self-assembled from cyclodextrin, photosensitizer, and prodrug Hyaluronic acid-Pyropheophobide and JQ1	Blockade of immunosuppression molecules; ROS-driven ICD; Local and systemic tumor inhibition; Enhancement of immunogenicity; Promote intertumoral infiltration of cytotoxic T lymphocytes	Subcutaneous and orthotopic Panc02 syngeneic pancreatic tumor model	256
Exosomal targeting of Notch pathway protein	Pancreatic cell-derived exosomal NPs	Decreased Notch signaling and mitochondria-dependent apoptosis; *In vitro* inhibition of human PC cell line growth	Various human PC cell lines	365
Nanocarrier enhanced delivery and reduced toxicity	PEG-PLGA NPs encapsulating ICD inducer oxaliplatin	Induced IFNγ expressing tumor-infiltrating CD8 T cell	Subcutaneous Panc02 syngeneic pancreatic tumor model	366

## Abbreviations

APC: antigen-presenting cells
ATF: amino-terminal fragment
AuNPs: gold nanoparticles
BBB: blood-brain barrier
CAF: cancer-associated fibroblast
CAR-T cell: chimeric antigen receptor T cell
DEX: dexamethasone
DOX: doxorubicin
DSPE: 1,2-distearoyl-sn-glycerol-3 phosphoethanolamine-N-amino
DTX: docetaxel
ECM: extracellular matrix
EMA: european medicine agency
EMT: epithelial to mesenchymal transition
FDA: food and drug administration
FOLFIRINOX: folinic acid, 5-fluorouracil, irinotecan, and oxaliplatin
GEM: gemcitabine
IFP: interstitial fluid pressure
KRAS: kirsten rat sarcoma viral oncogene homolog
mCRPC: metastatic-castration resistance prostate cancer
MnMEIO: manganese-doped magnetism-engineered iron oxide
MRI: magnetic resonance imaging
MSC: mesenchymal stem cells
MSN: mesoporous silica nanoparticle
MTAI: microwave induced thermoacoustic imaging
Nab-PTX: nano-albumin bound-paclitaxel
NIRF: near-infrared fluorescence imaging
NK cell: natural killer cell
NP: nanoparticle
NSCLC: non-small cell lung cancer
OX: oxaliplatin
PC: pancreatic cancer
PD-1: programmed cell death receptor-1
PDAC: pancreatic ductal adenocarcinoma
PDEAEM: poly(diethylaminoethylmethacrylate)
PDT: photodynamic therapy
PEG: polyethylene glycol
PGA: poly(L-glutamic acid)
PLA: polylactic acid
PLGH: poly (DL-lactide-co-glycolide)
PTT: photothermal therapy
RIG-1: retinoic acid-inducible gene-1
SHH: sonic hedgehog
SPION: superparamagnetic iron oxide nanoparticles
TAM: tumor-associated macrophage
TME: tumor microenvironment
t-PA: tissue plasminogen activator derived peptides
uPAR: urokinase plasminogen activator receptor
